# Supplementary figures and images for: Immune training enhances anti-viral responses and improves outcomes in Pax5−/+ mice susceptible to chronic infection
Source: EMBO Mol Med. 2025 Mar 13;17(4):696–721. doi: 10.1038/s44321-025-00208-4 (PMC11982562; doi:10.1038/s44321-025-00208-4)

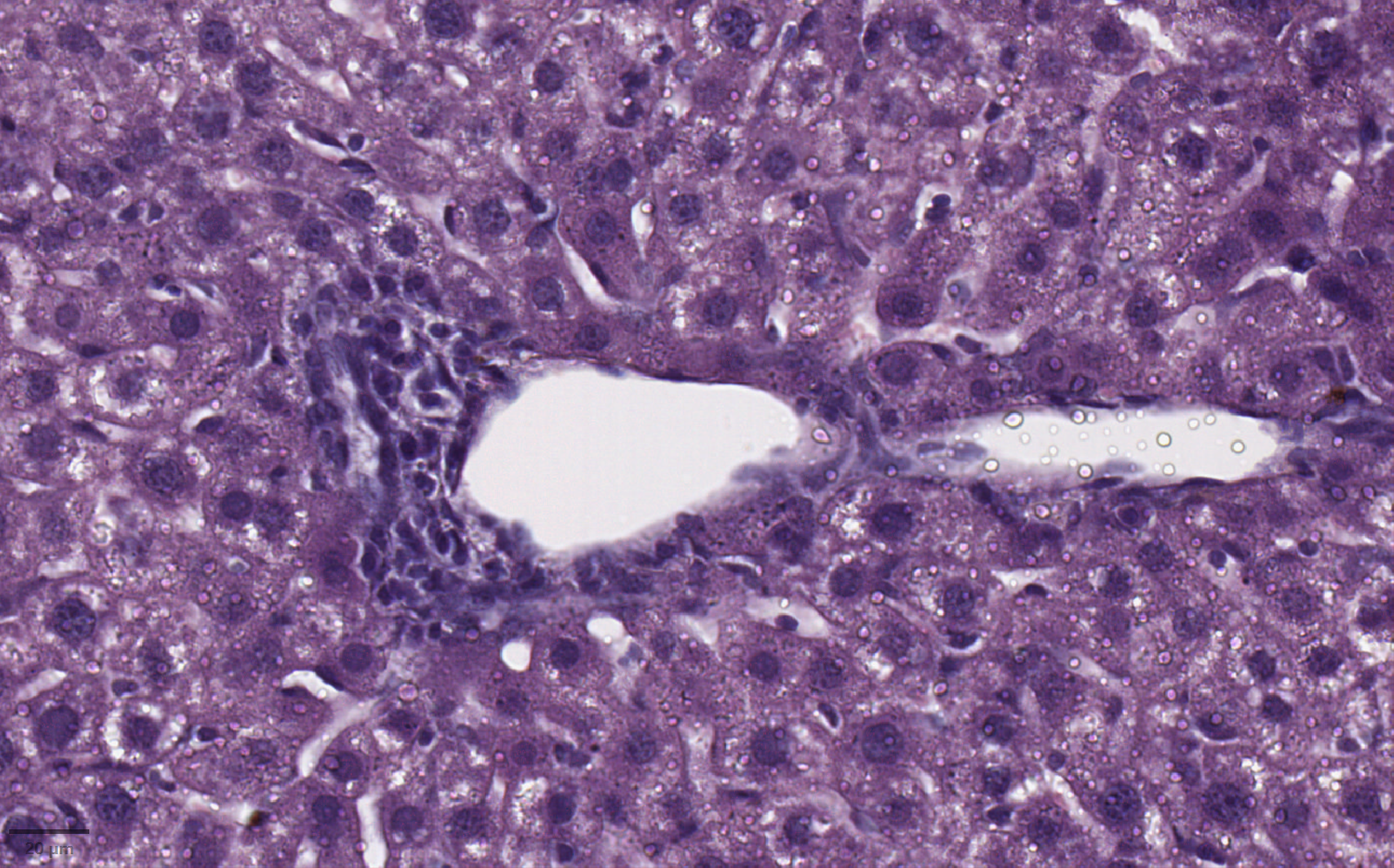

Supplement: Supplementary file 4 — Source data Fig. 2 [file 44321_2025_208_MOESM4_ESM.zip › Figure2/2E/H&E graph/Pax5 Day120 round.tif]

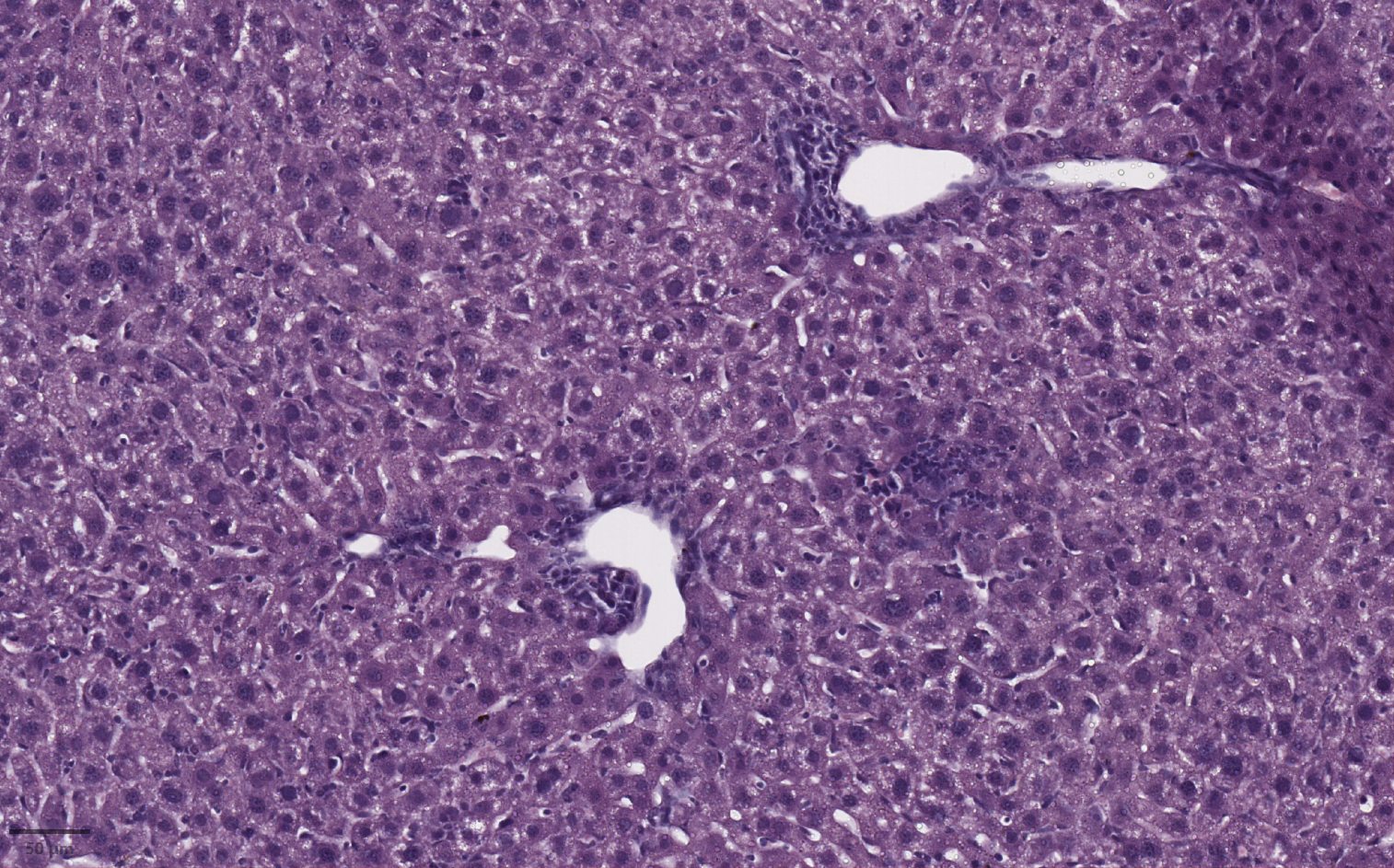

Supplement: Supplementary file 4 — Source data Fig. 2 [file 44321_2025_208_MOESM4_ESM.zip › Figure2/2E/H&E graph/Pax5 Day120.tif]

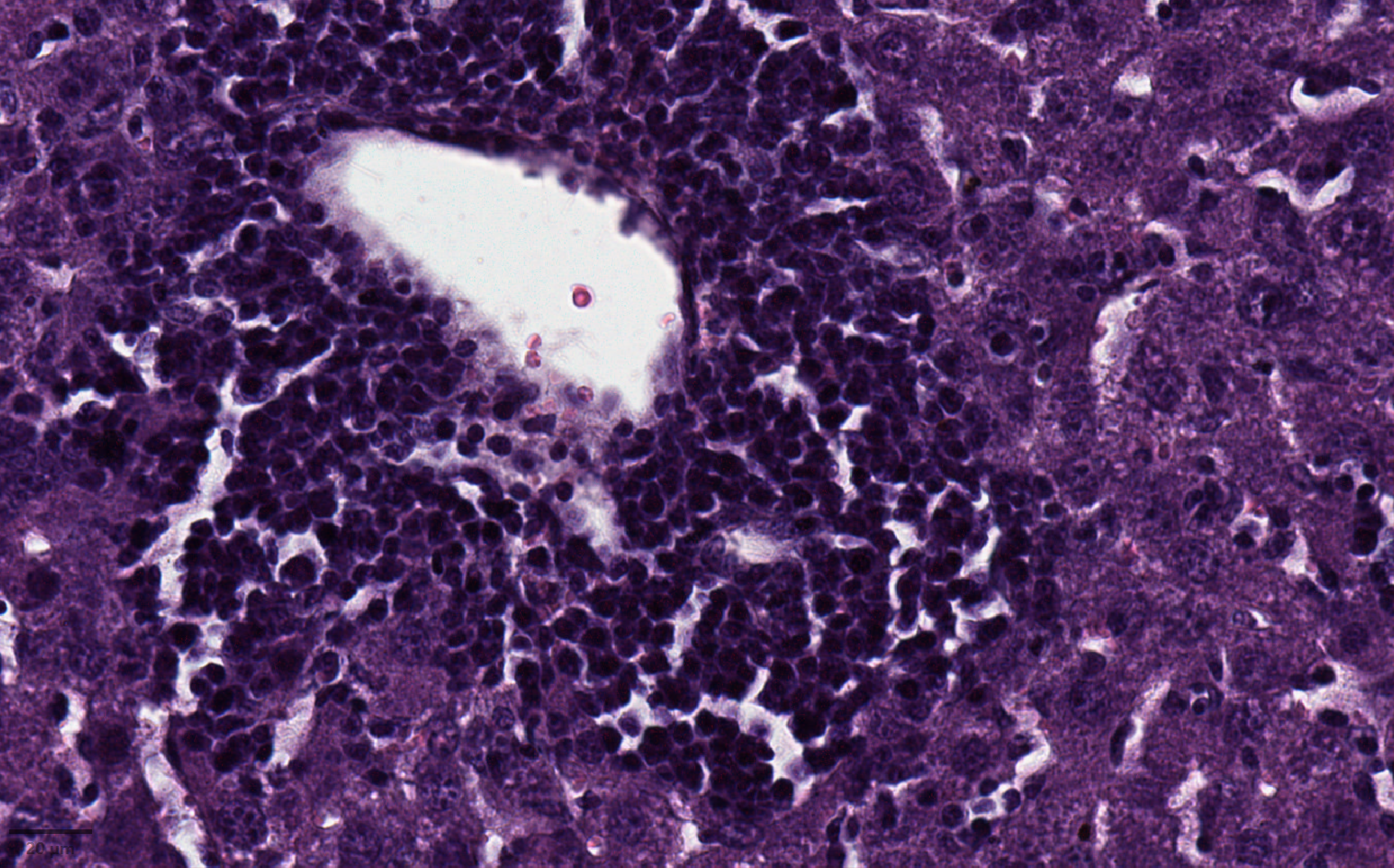

Supplement: Supplementary file 4 — Source data Fig. 2 [file 44321_2025_208_MOESM4_ESM.zip › Figure2/2E/H&E graph/Pax5 Day15 round.tif]

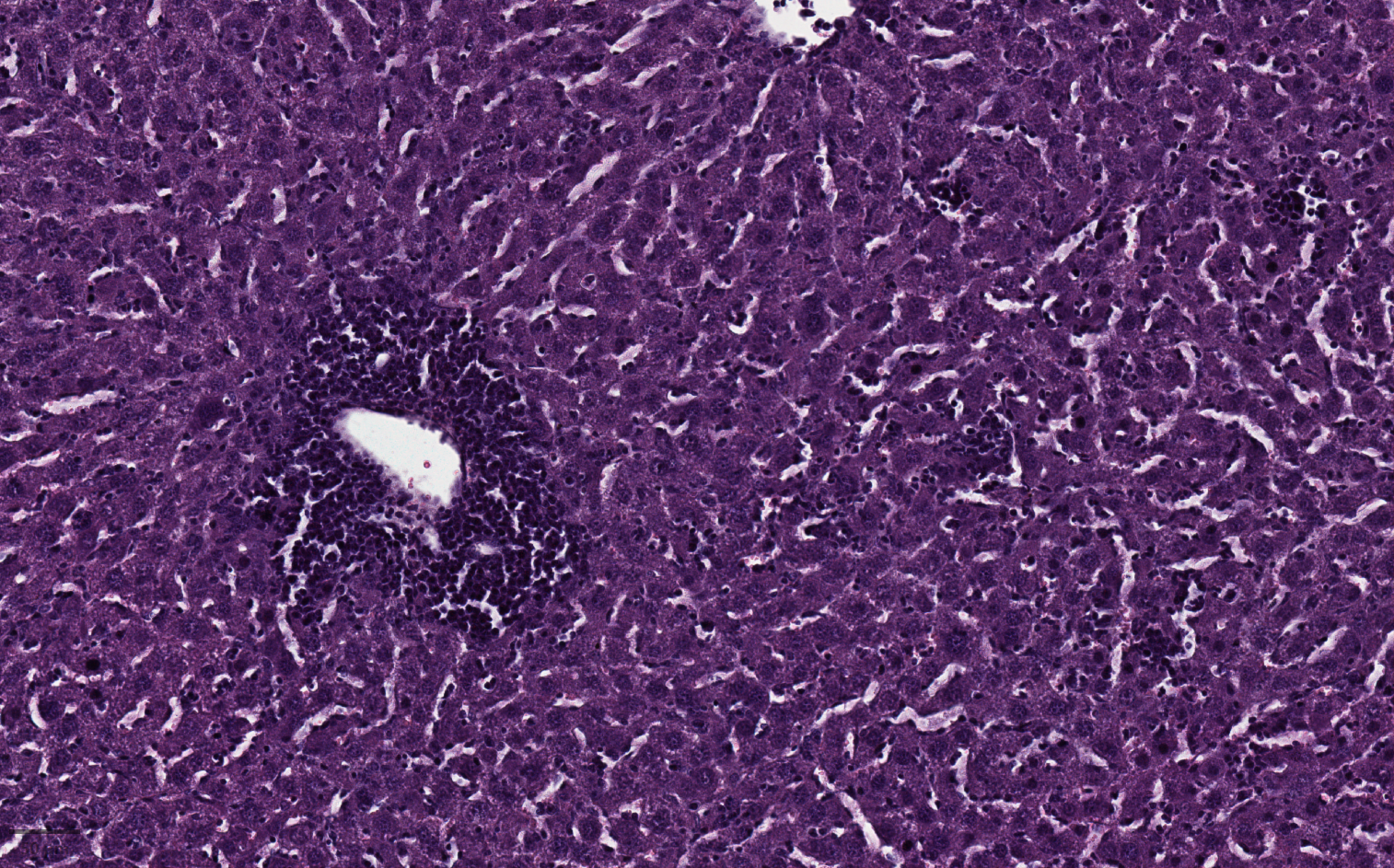

Supplement: Supplementary file 4 — Source data Fig. 2 [file 44321_2025_208_MOESM4_ESM.zip › Figure2/2E/H&E graph/Pax5 Day15.tif]

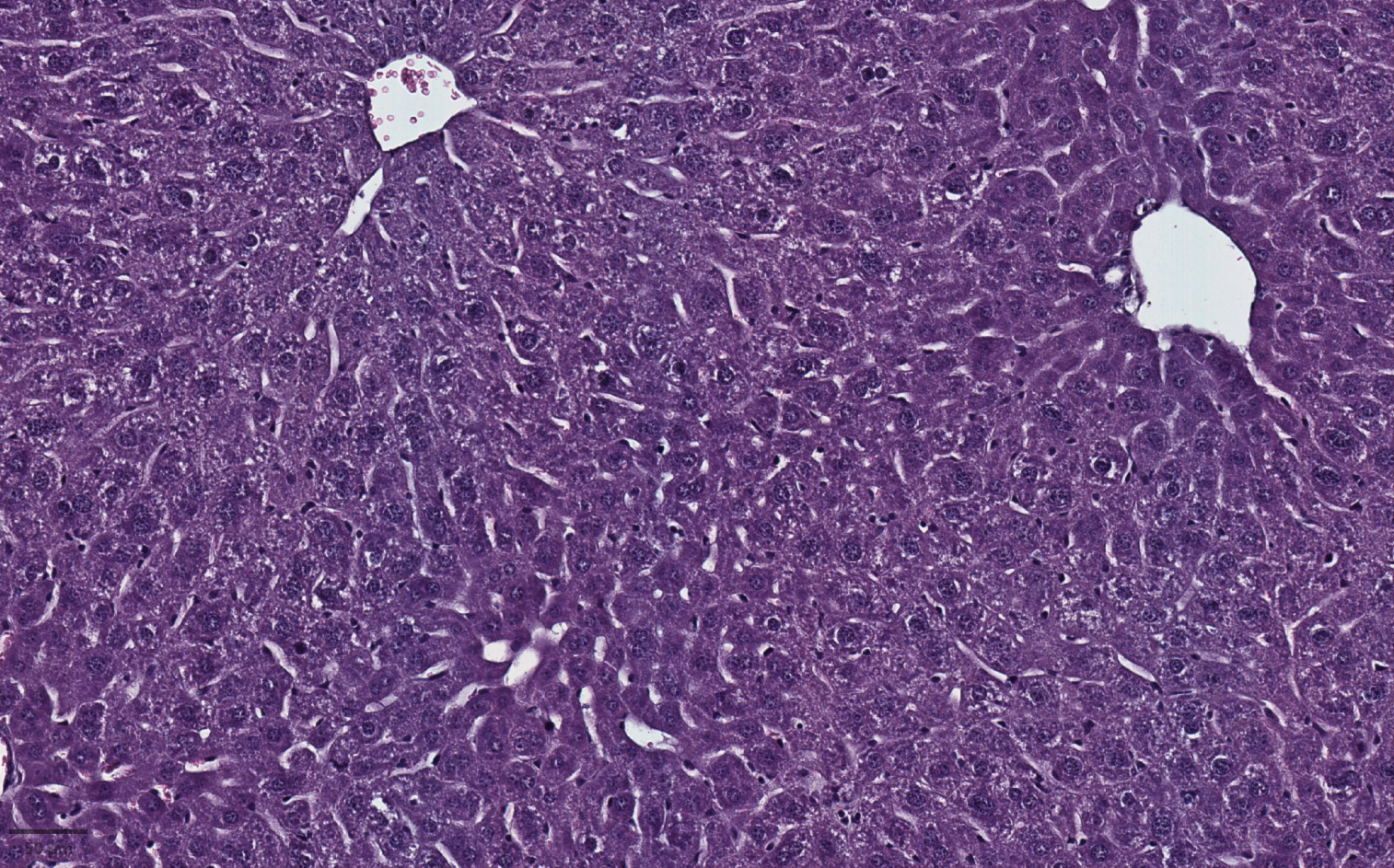

Supplement: Supplementary file 4 — Source data Fig. 2 [file 44321_2025_208_MOESM4_ESM.zip › Figure2/2E/H&E graph/Pax5 Naive.tif]

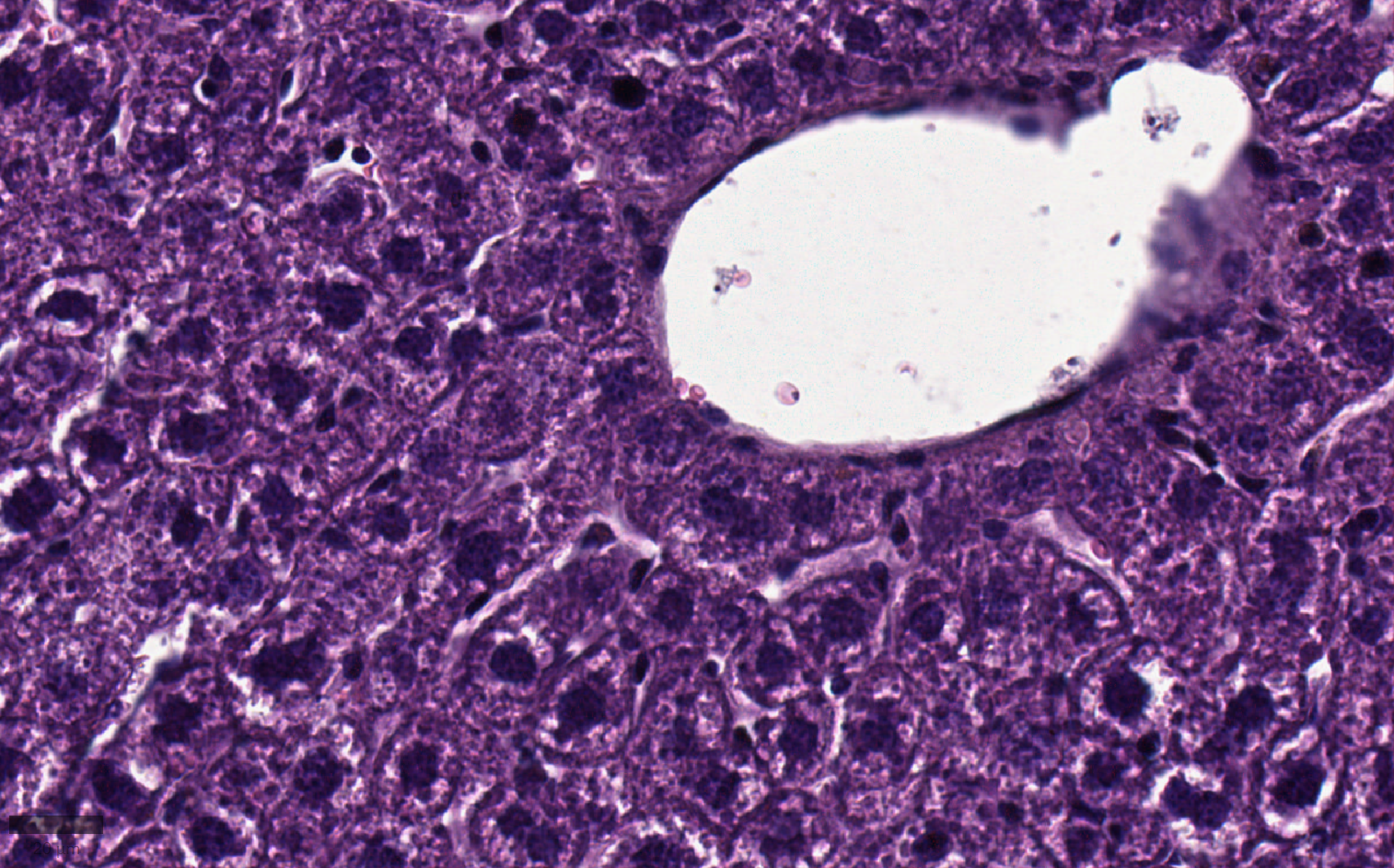

Supplement: Supplementary file 4 — Source data Fig. 2 [file 44321_2025_208_MOESM4_ESM.zip › Figure2/2E/H&E graph/WT Day120 round.tif]

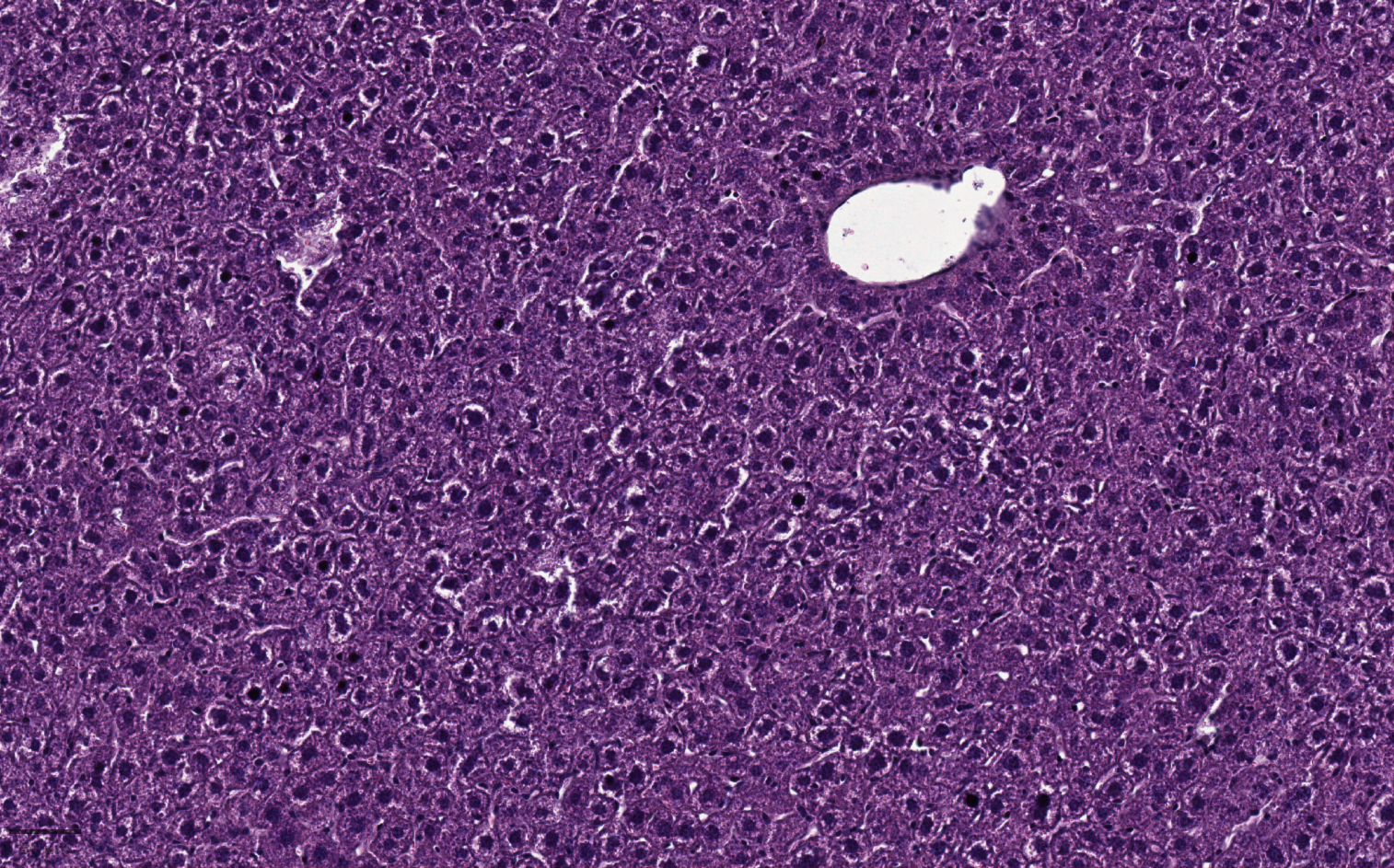

Supplement: Supplementary file 4 — Source data Fig. 2 [file 44321_2025_208_MOESM4_ESM.zip › Figure2/2E/H&E graph/WT Day120.tif]

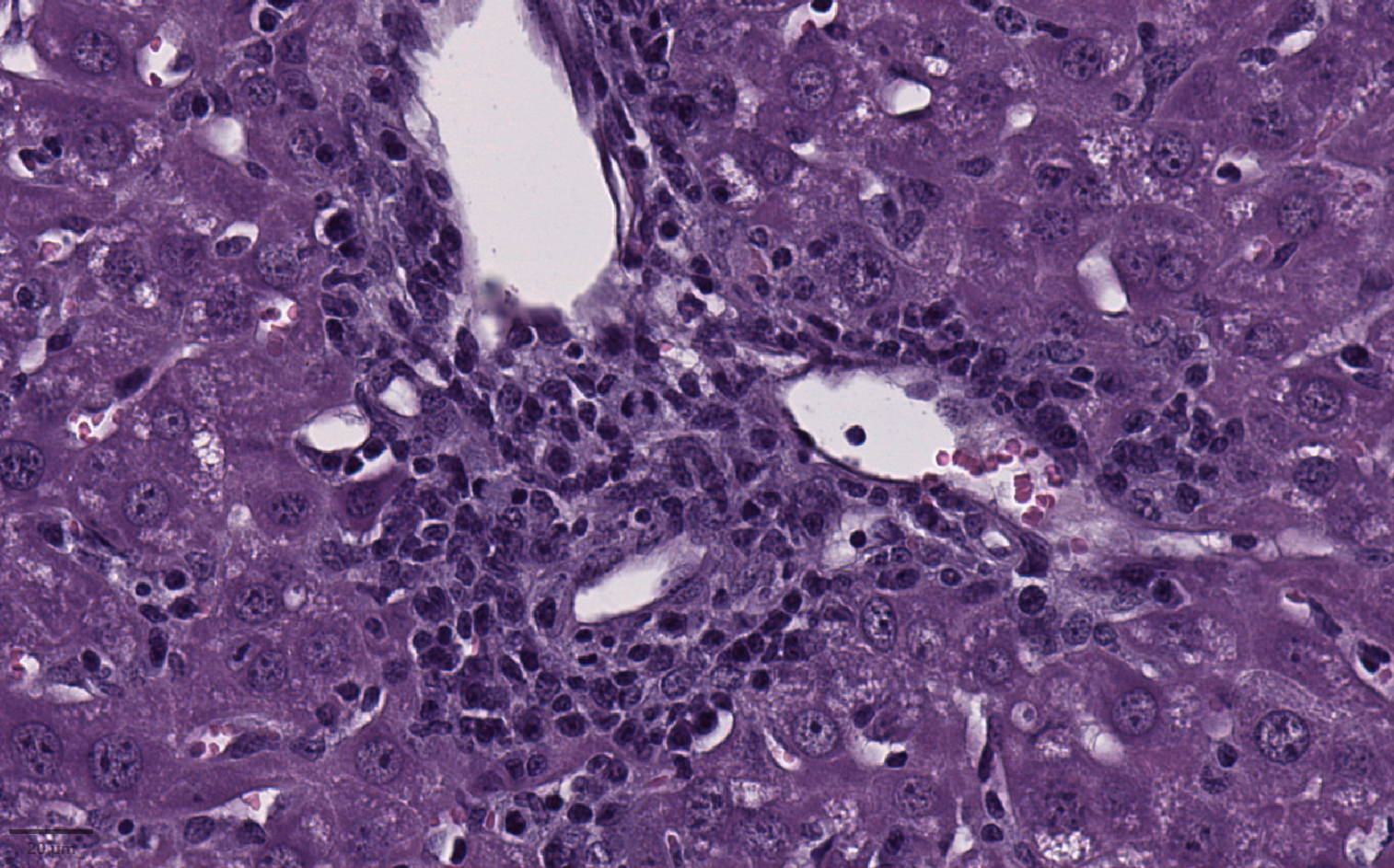

Supplement: Supplementary file 4 — Source data Fig. 2 [file 44321_2025_208_MOESM4_ESM.zip › Figure2/2E/H&E graph/WT Day15 round.tif]

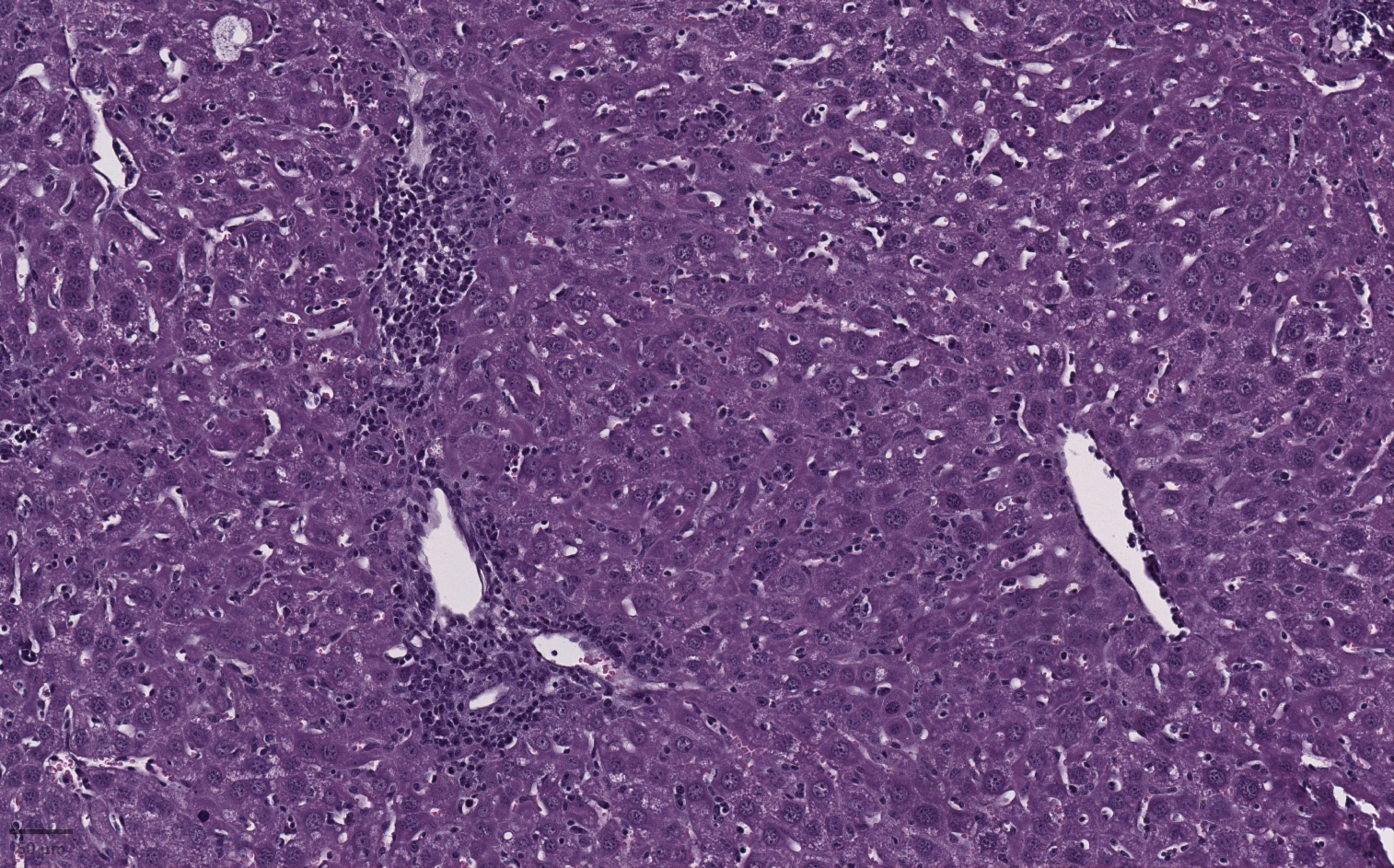

Supplement: Supplementary file 4 — Source data Fig. 2 [file 44321_2025_208_MOESM4_ESM.zip › Figure2/2E/H&E graph/WT Day15.tif]

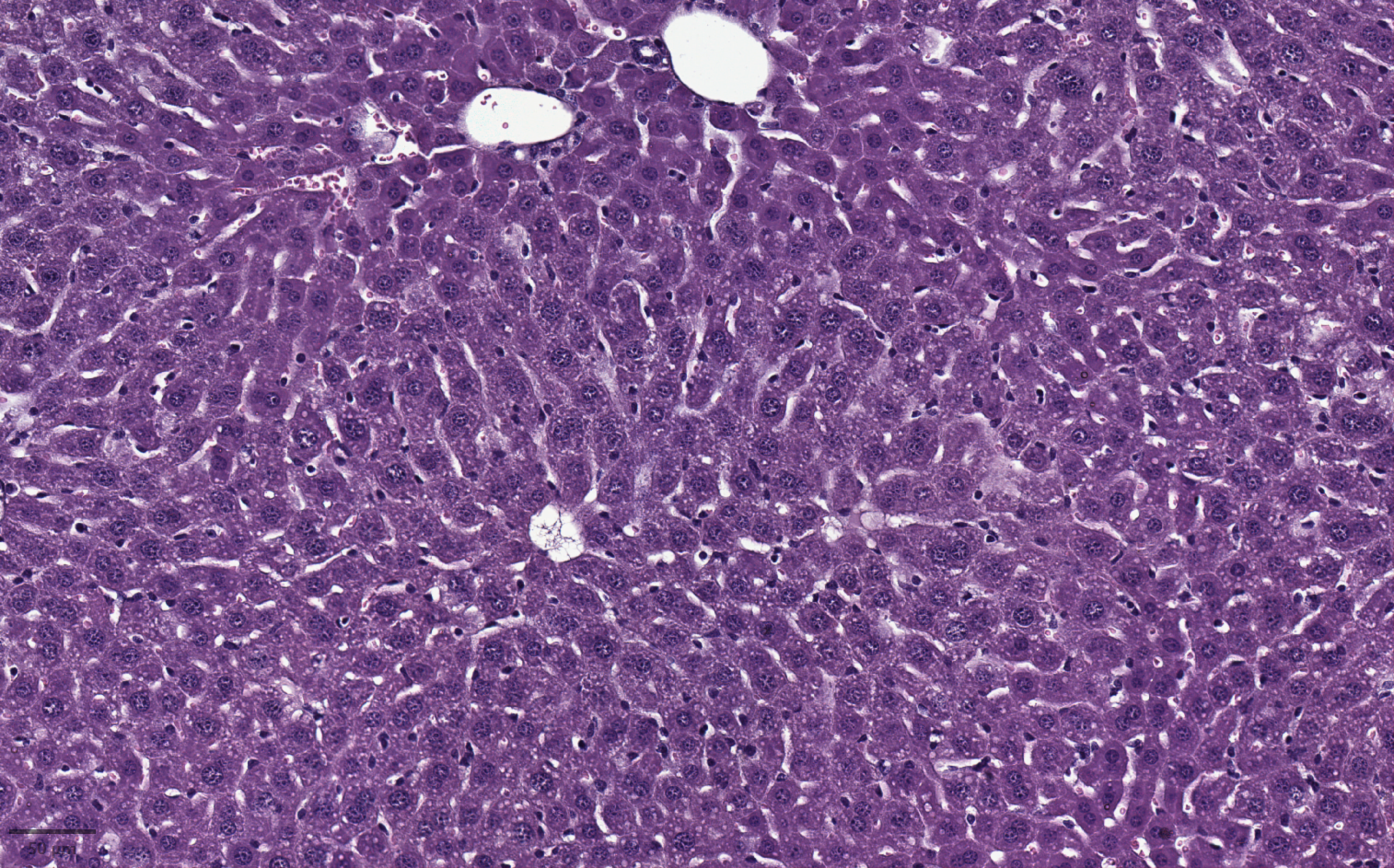

Supplement: Supplementary file 4 — Source data Fig. 2 [file 44321_2025_208_MOESM4_ESM.zip › Figure2/2E/H&E graph/WT Naive.tif]

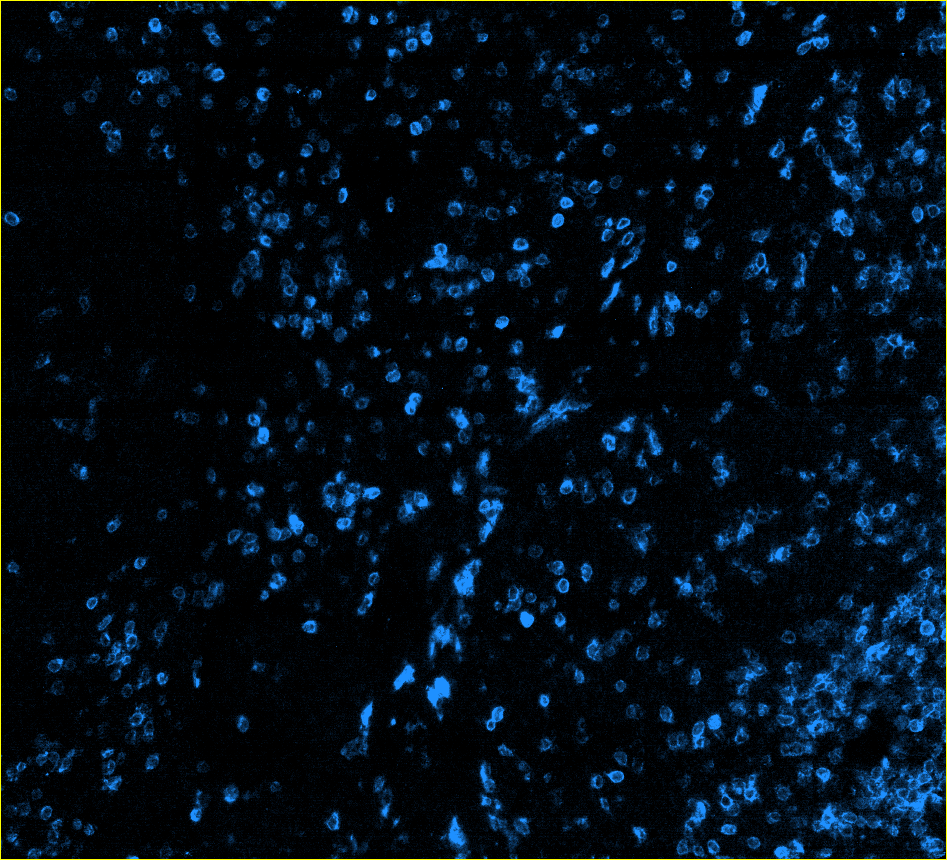

Supplement: Supplementary file 9 — Source data Fig. 7 [file 44321_2025_208_MOESM9_ESM.zip › Figure7/7J/LCMV/cd3.tif]

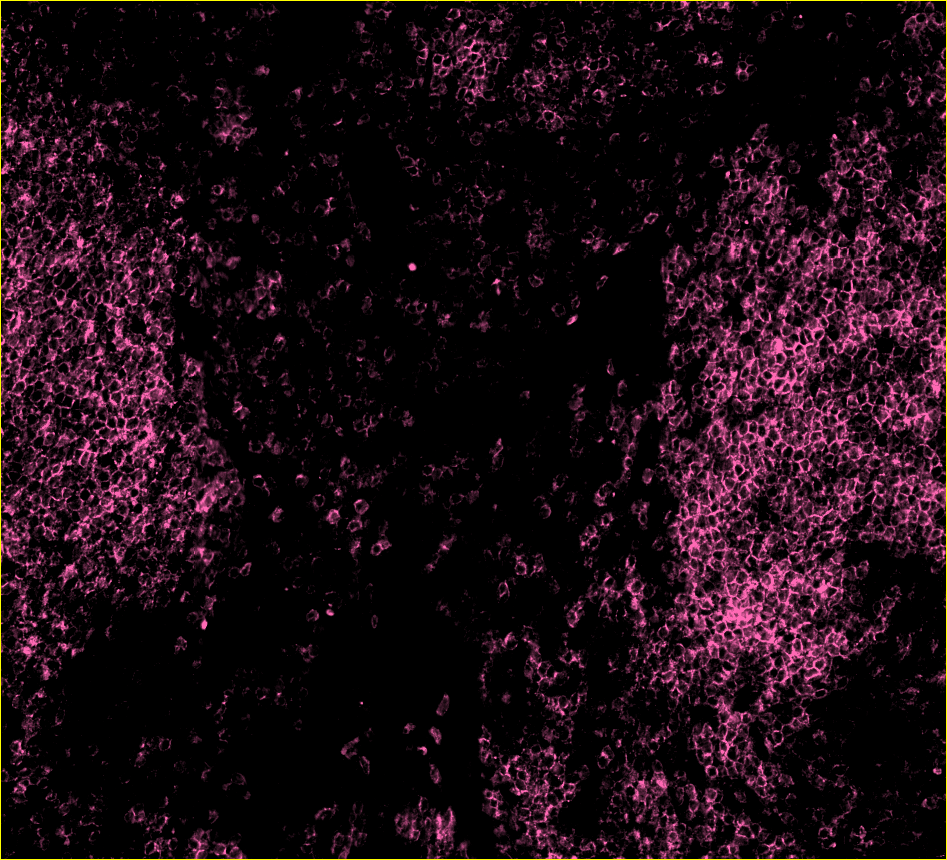

Supplement: Supplementary file 9 — Source data Fig. 7 [file 44321_2025_208_MOESM9_ESM.zip › Figure7/7J/LCMV/b220.tif]

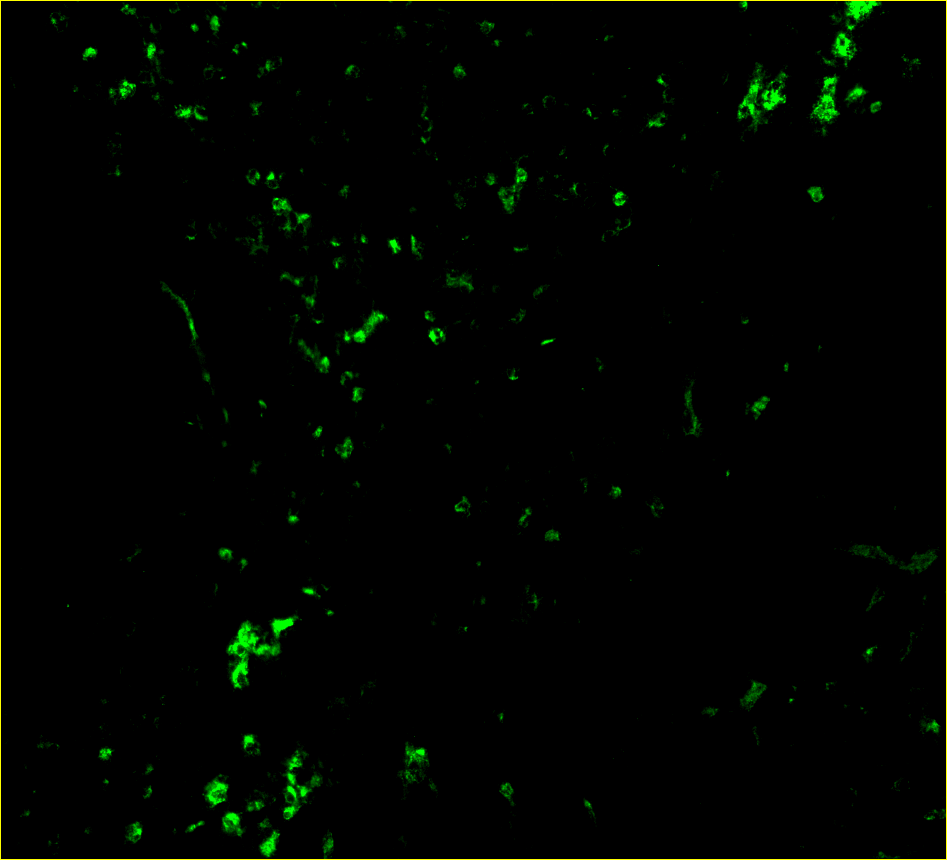

Supplement: Supplementary file 9 — Source data Fig. 7 [file 44321_2025_208_MOESM9_ESM.zip › Figure7/7J/LCMV/ly6c.tif]

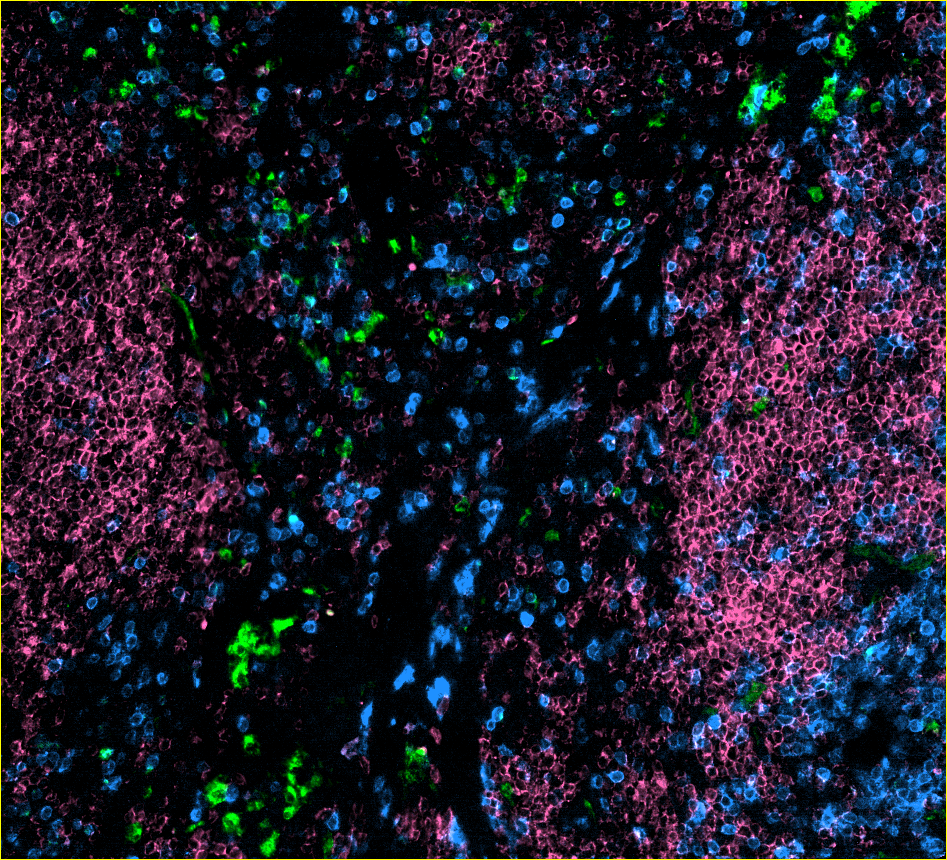

Supplement: Supplementary file 9 — Source data Fig. 7 [file 44321_2025_208_MOESM9_ESM.zip › Figure7/7J/LCMV/merge.tif]

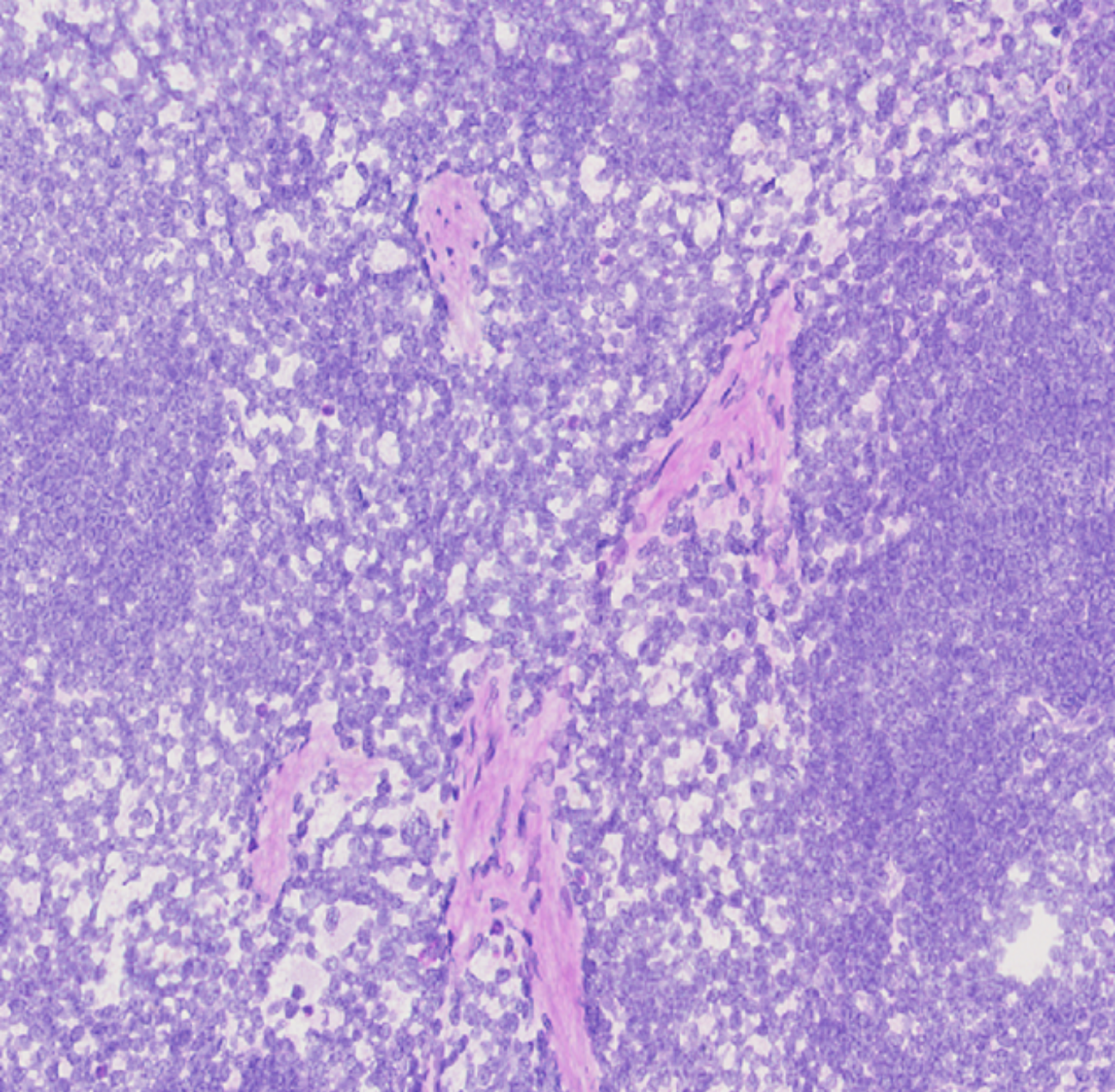

Supplement: Supplementary file 9 — Source data Fig. 7 [file 44321_2025_208_MOESM9_ESM.zip › Figure7/7J/LCMV/H&E.tiff]

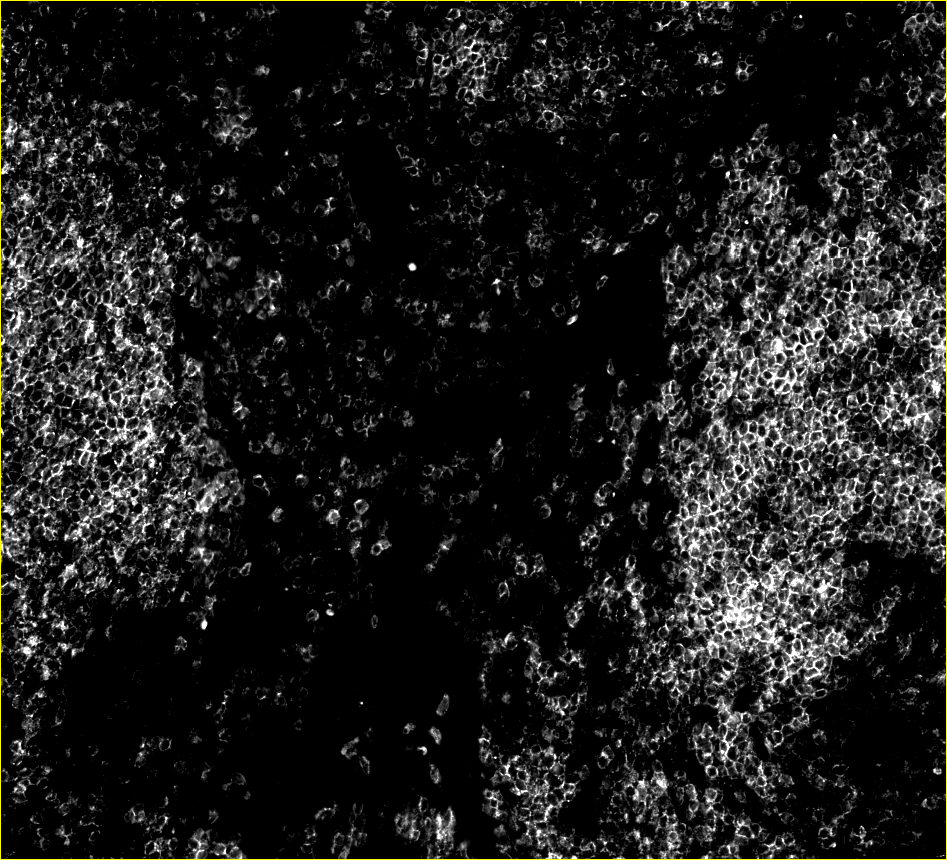

Supplement: Supplementary file 9 — Source data Fig. 7 [file 44321_2025_208_MOESM9_ESM.zip › Figure7/7J/LCMV/grayscale/b220grey.tif]

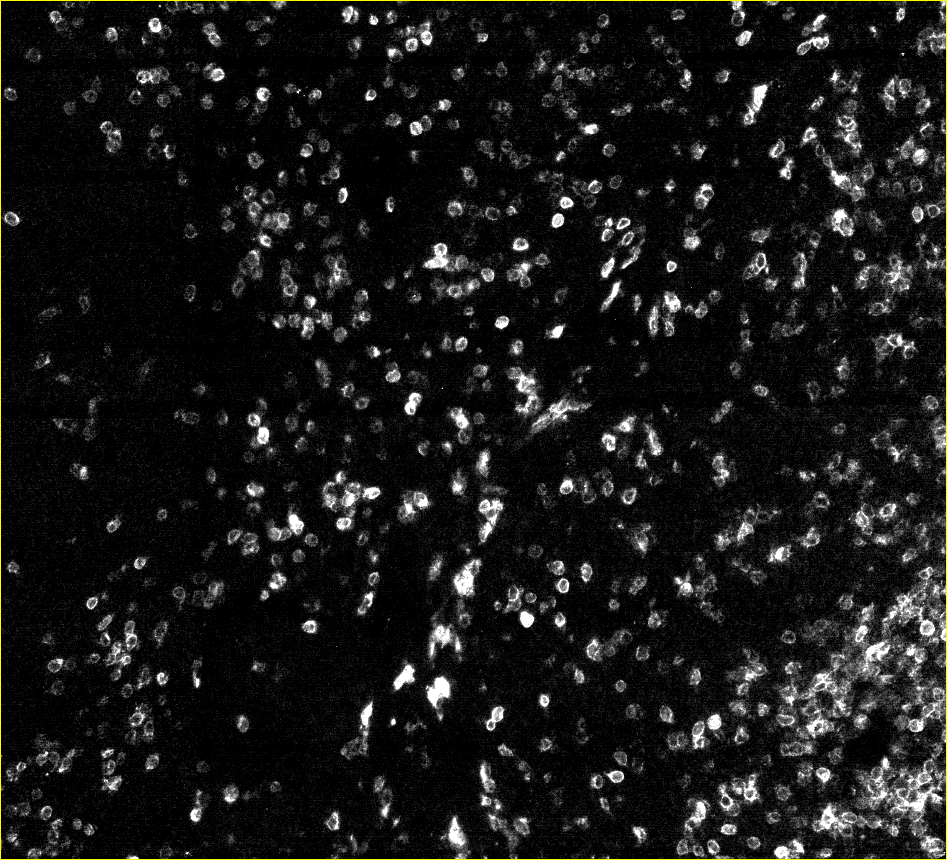

Supplement: Supplementary file 9 — Source data Fig. 7 [file 44321_2025_208_MOESM9_ESM.zip › Figure7/7J/LCMV/grayscale/cd3grey.tif]

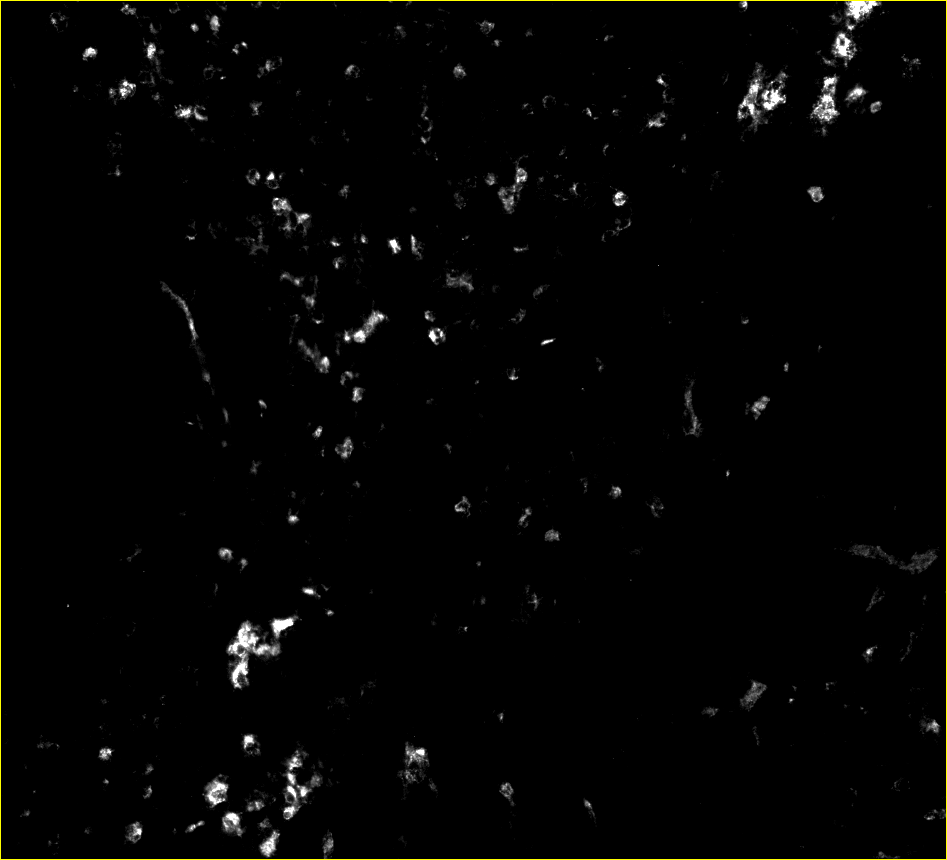

Supplement: Supplementary file 9 — Source data Fig. 7 [file 44321_2025_208_MOESM9_ESM.zip › Figure7/7J/LCMV/grayscale/ly6cgrey.tif]

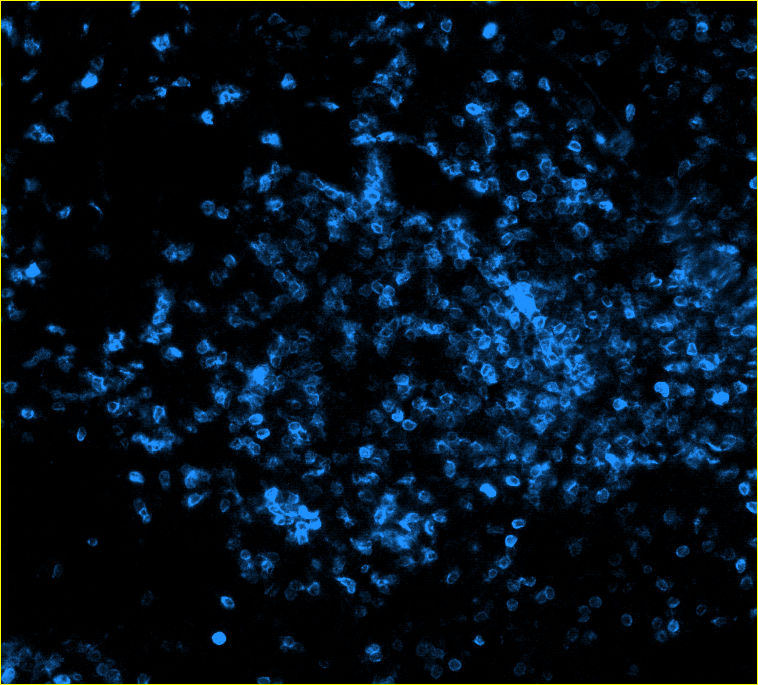

Supplement: Supplementary file 9 — Source data Fig. 7 [file 44321_2025_208_MOESM9_ESM.zip › Figure7/7J/bglucan, LCMV/cd3.tif]

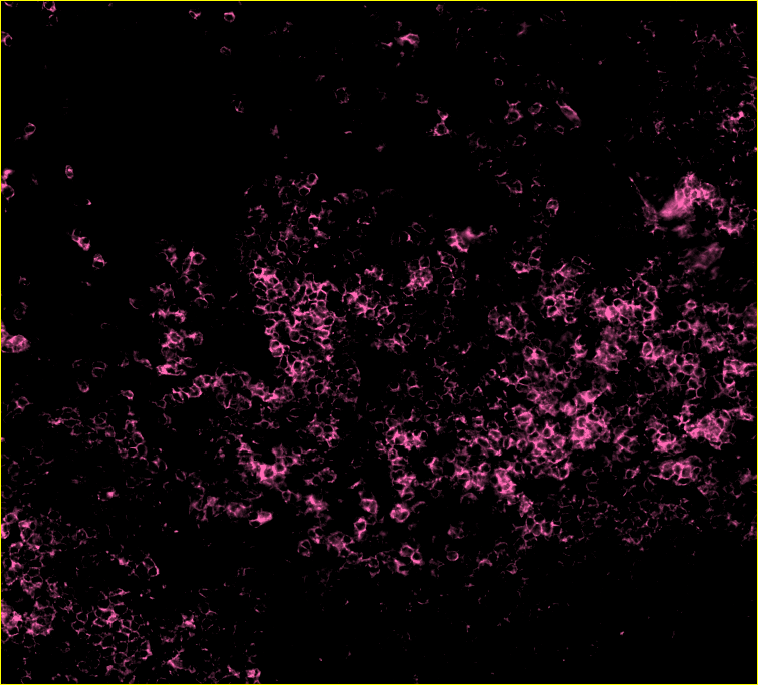

Supplement: Supplementary file 9 — Source data Fig. 7 [file 44321_2025_208_MOESM9_ESM.zip › Figure7/7J/bglucan, LCMV/b220.tif]

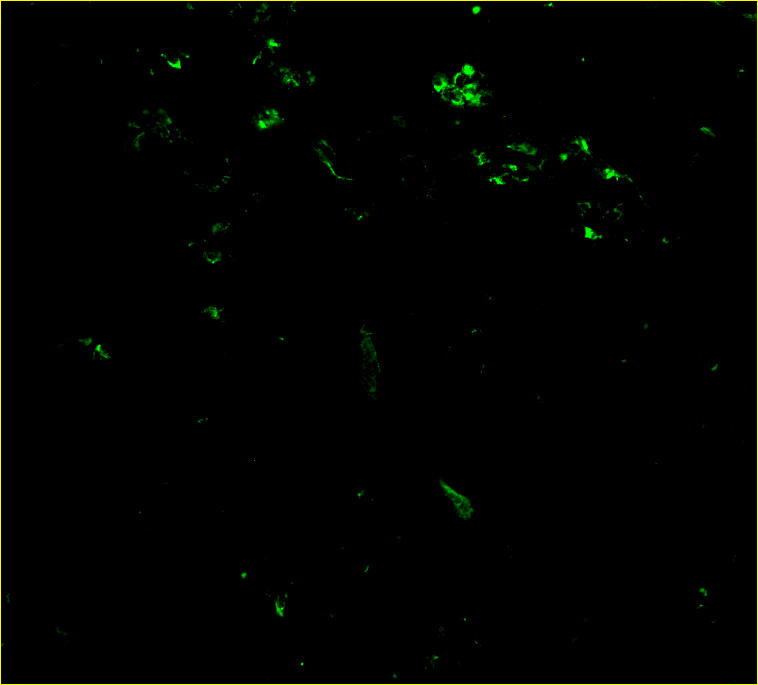

Supplement: Supplementary file 9 — Source data Fig. 7 [file 44321_2025_208_MOESM9_ESM.zip › Figure7/7J/bglucan, LCMV/ly6c.tif]

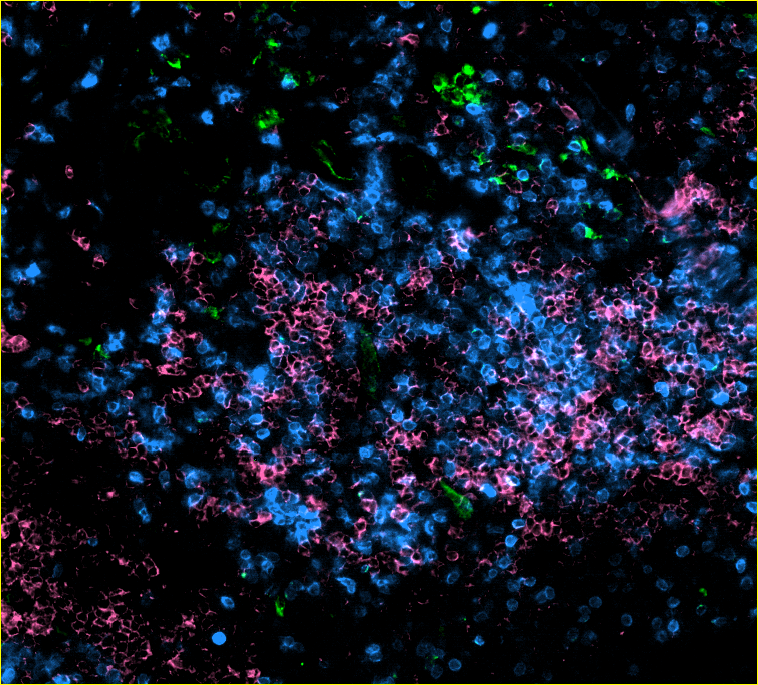

Supplement: Supplementary file 9 — Source data Fig. 7 [file 44321_2025_208_MOESM9_ESM.zip › Figure7/7J/bglucan, LCMV/merge.tif]

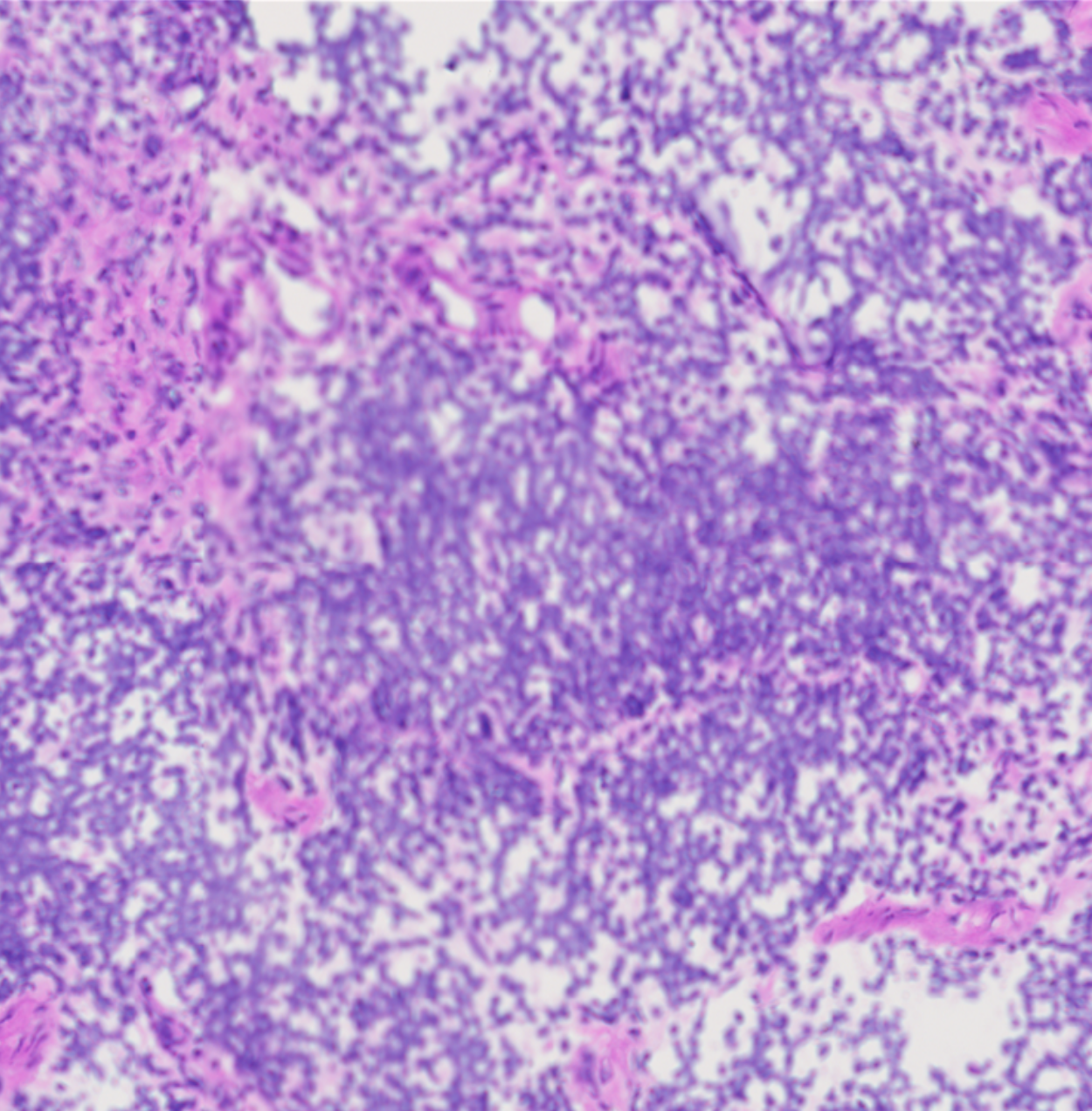

Supplement: Supplementary file 9 — Source data Fig. 7 [file 44321_2025_208_MOESM9_ESM.zip › Figure7/7J/bglucan, LCMV/H&E.tiff]

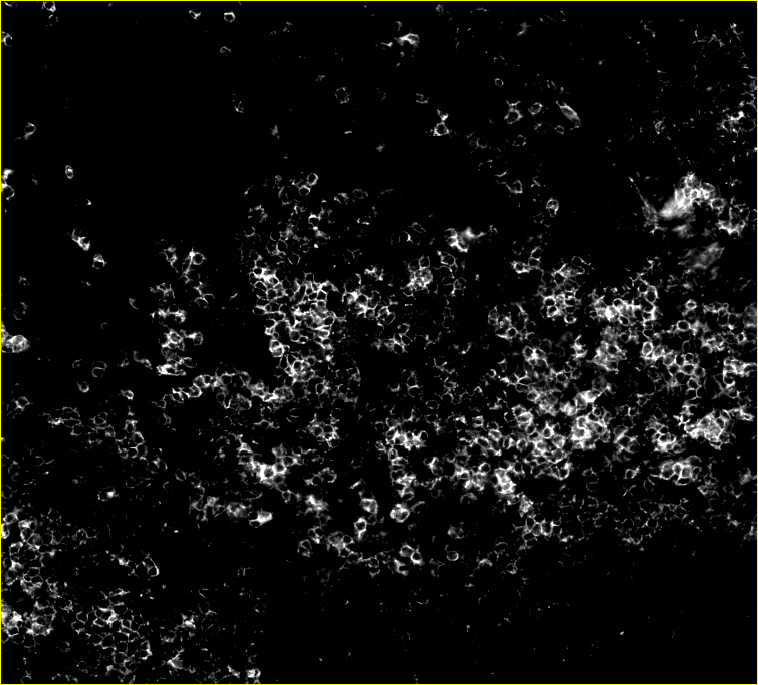

Supplement: Supplementary file 9 — Source data Fig. 7 [file 44321_2025_208_MOESM9_ESM.zip › Figure7/7J/bglucan, LCMV/grayscale/b220grey.tif]

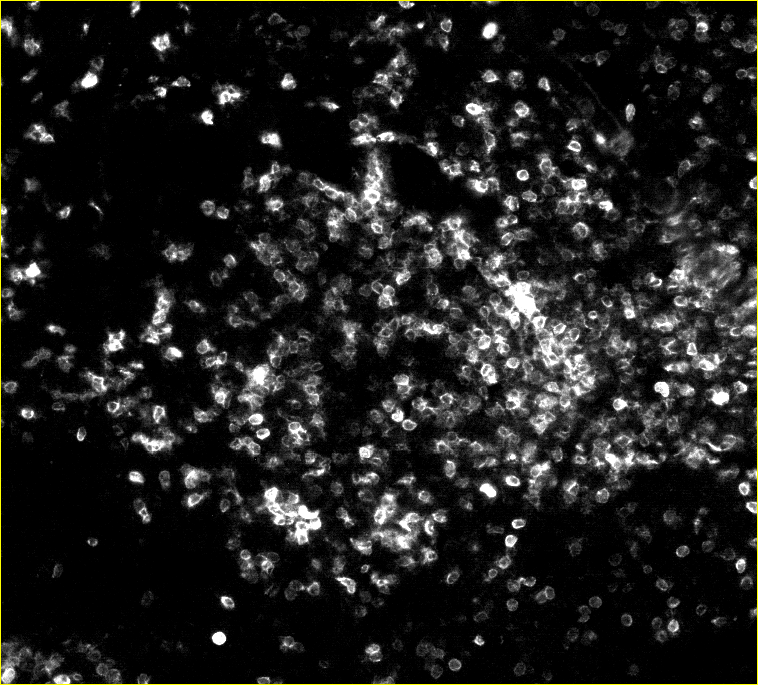

Supplement: Supplementary file 9 — Source data Fig. 7 [file 44321_2025_208_MOESM9_ESM.zip › Figure7/7J/bglucan, LCMV/grayscale/cd3grey.tif]

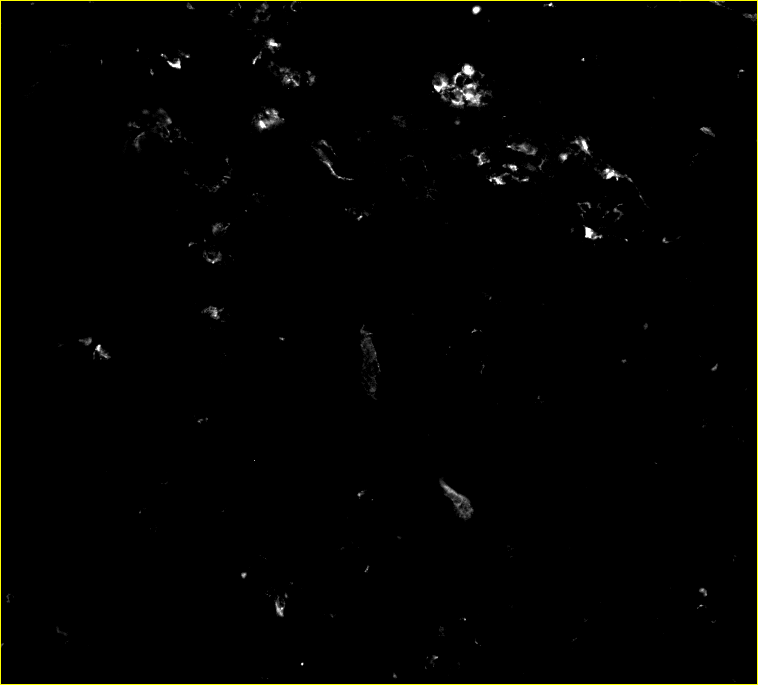

Supplement: Supplementary file 9 — Source data Fig. 7 [file 44321_2025_208_MOESM9_ESM.zip › Figure7/7J/bglucan, LCMV/grayscale/ly6cgrey.tif]

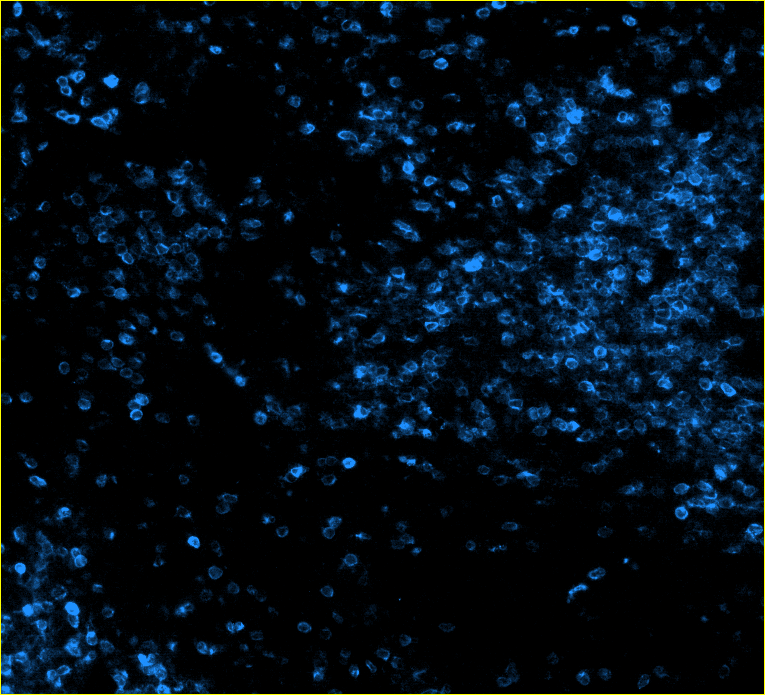

Supplement: Supplementary file 9 — Source data Fig. 7 [file 44321_2025_208_MOESM9_ESM.zip › Figure7/7J/LCMV, bglucan/cd3.tif]

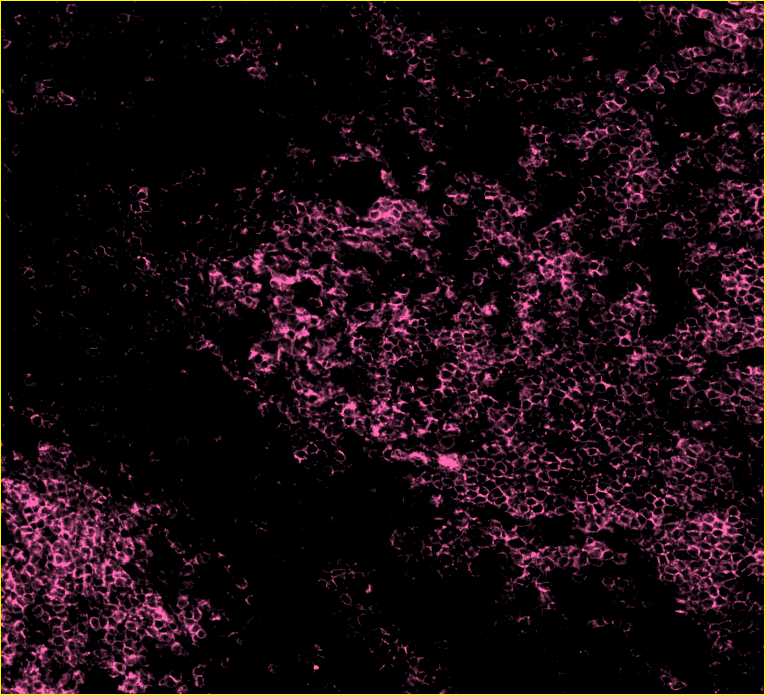

Supplement: Supplementary file 9 — Source data Fig. 7 [file 44321_2025_208_MOESM9_ESM.zip › Figure7/7J/LCMV, bglucan/b220.tif]

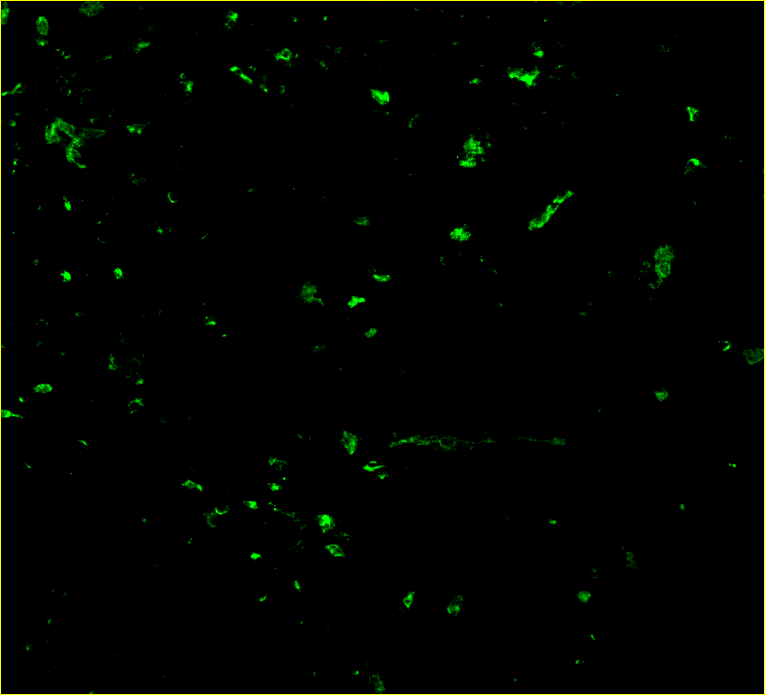

Supplement: Supplementary file 9 — Source data Fig. 7 [file 44321_2025_208_MOESM9_ESM.zip › Figure7/7J/LCMV, bglucan/ly6c.tif]

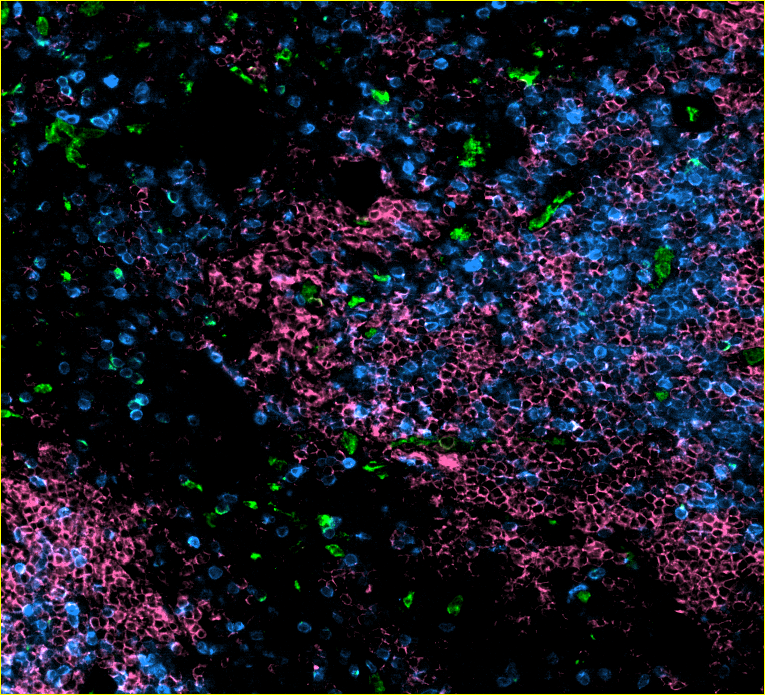

Supplement: Supplementary file 9 — Source data Fig. 7 [file 44321_2025_208_MOESM9_ESM.zip › Figure7/7J/LCMV, bglucan/merge.tif]

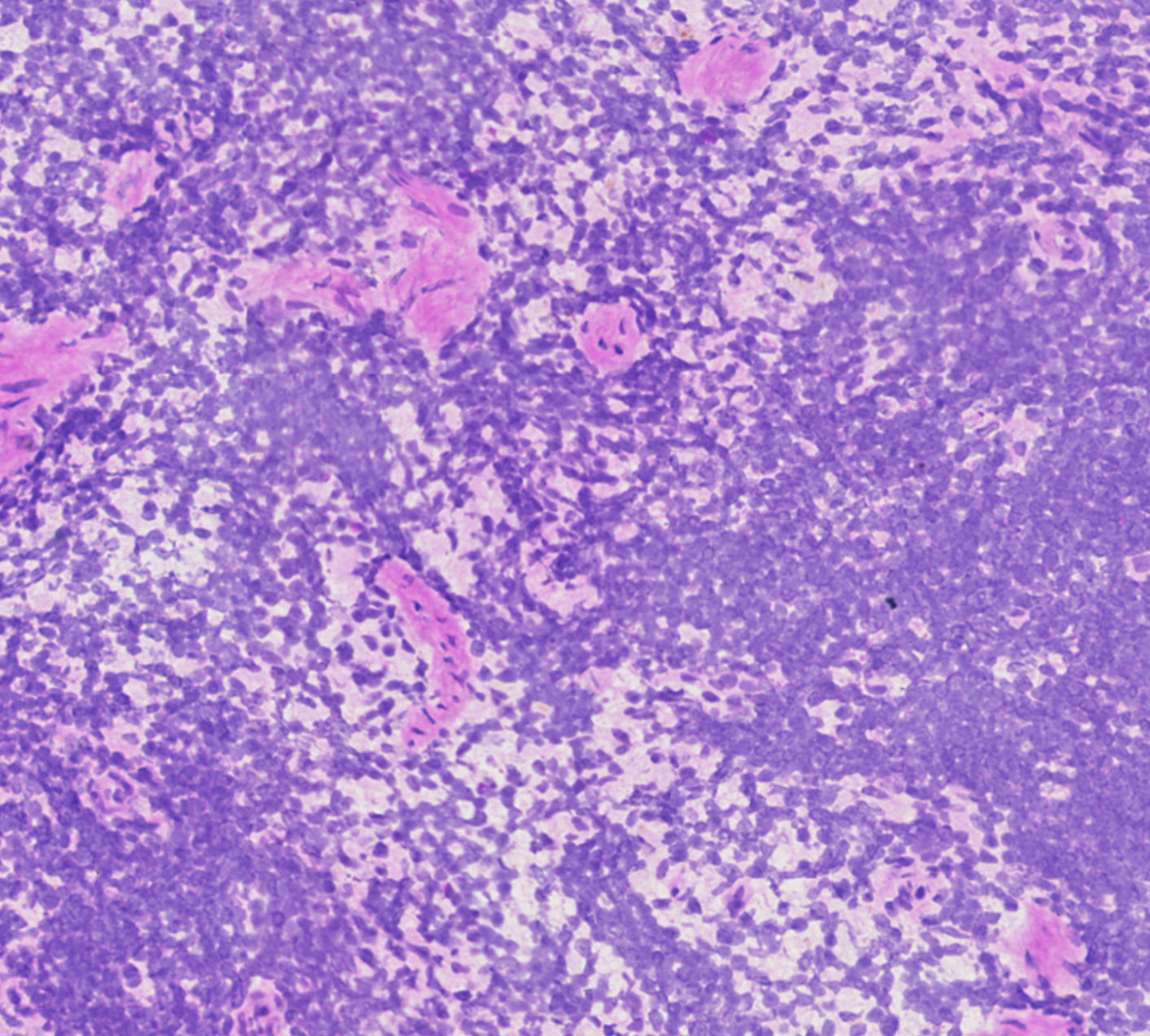

Supplement: Supplementary file 9 — Source data Fig. 7 [file 44321_2025_208_MOESM9_ESM.zip › Figure7/7J/LCMV, bglucan/H&E.tiff]

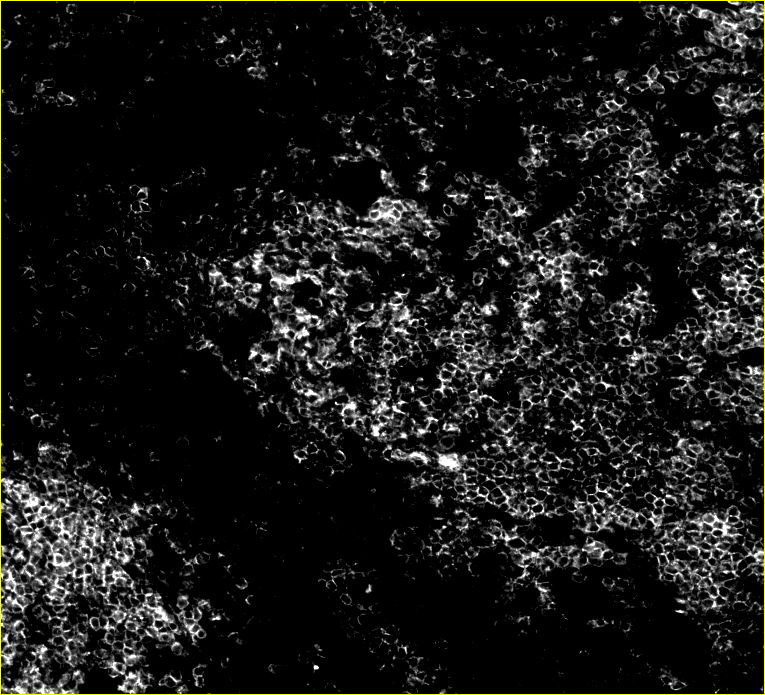

Supplement: Supplementary file 9 — Source data Fig. 7 [file 44321_2025_208_MOESM9_ESM.zip › Figure7/7J/LCMV, bglucan/grayscale/b220grey.tif]

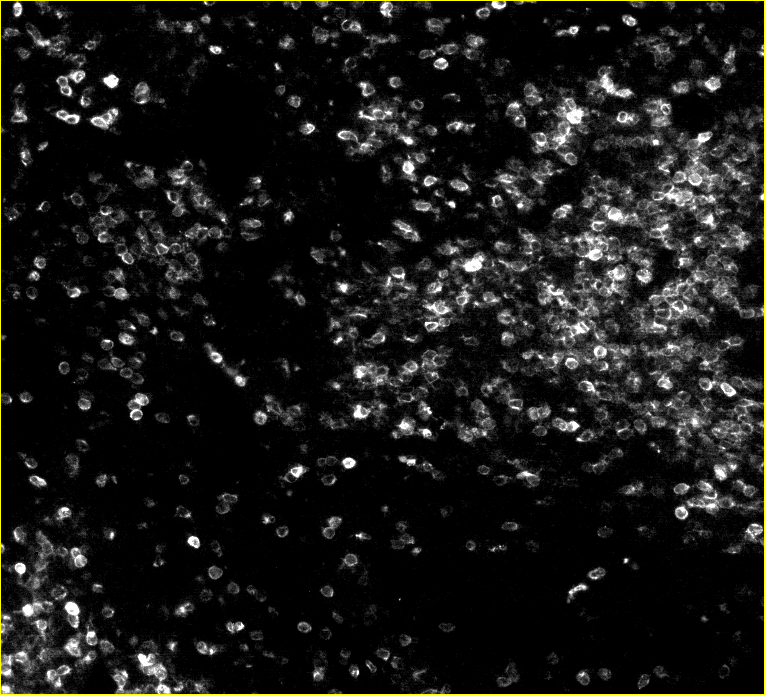

Supplement: Supplementary file 9 — Source data Fig. 7 [file 44321_2025_208_MOESM9_ESM.zip › Figure7/7J/LCMV, bglucan/grayscale/cd3grey.tif]

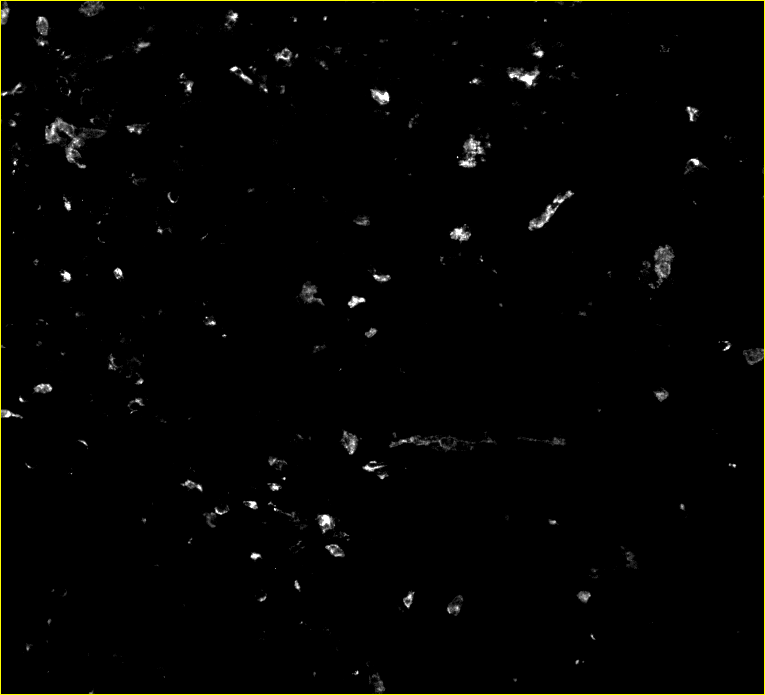

Supplement: Supplementary file 9 — Source data Fig. 7 [file 44321_2025_208_MOESM9_ESM.zip › Figure7/7J/LCMV, bglucan/grayscale/l6cgrey.tif]
