# Supplementary material for: Immune training enhances anti-viral responses and improves outcomes in Pax5−/+ mice susceptible to chronic infection
Source: EMBO Mol Med. 2025 Mar 13;17(4):696–721. doi: 10.1038/s44321-025-00208-4 (PMC11982562; doi:10.1038/s44321-025-00208-4)
Supplement: Supplementary file 11 — Expanded View Figures [file 44321_2025_208_MOESM11_ESM.pdf]

Expanded View Figures

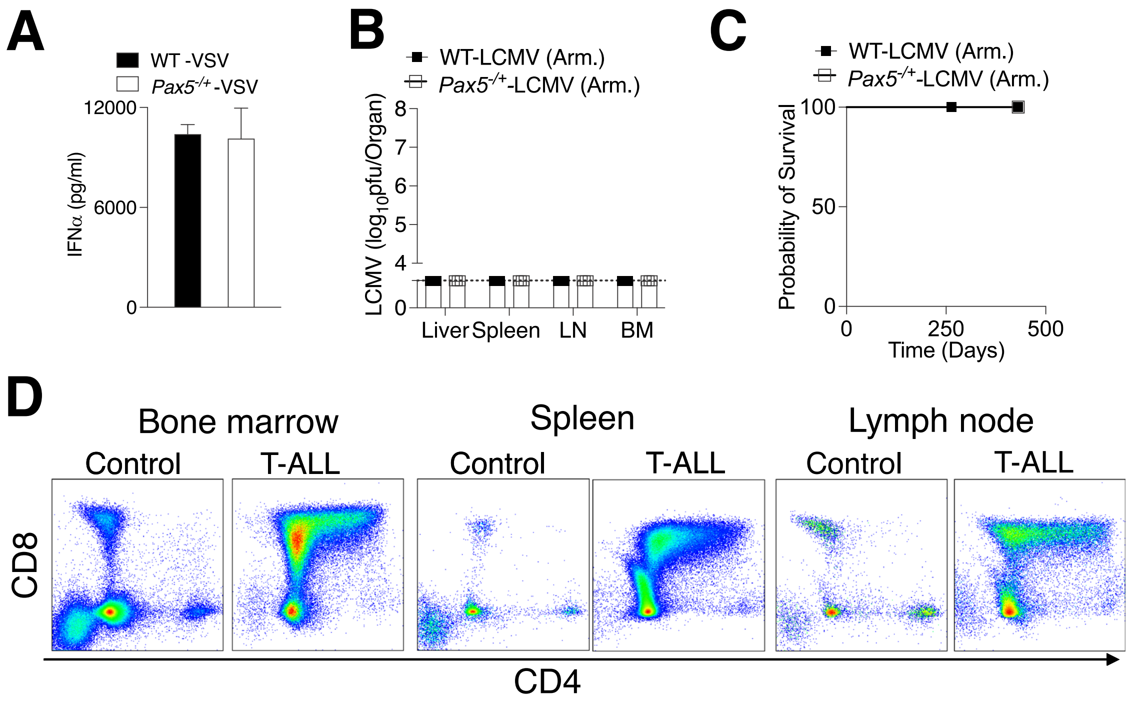

Figure EV1. Pax5 haploinsufficiency does not compromise hosts following VSV and acute LCMV infection.

(**A**) WT and *Pax5*$^{-/+}$ mice were infected with 10^6 PFU of VSV and IFN-α concentration was determined in the plasma of WT ($n = 3$ mice per group) and *Pax5*$^{+/-}$ mice ($n = 4$ mice per group) 24 h after infection. WT and *Pax5*$^{-/+}$ mice were infected with 10^6 PFU of LCMV-Armstrong strain (Arm.) and (**B**) viral titers were determined 15 days post infection using the plaque assay ($n = 3$ mice per group). Statistical analyses were performed using a Student´s *t* test (unpaired, two-tailed). (**C**) Survival was monitored ($n = 3$ mice per group). Statistical analysis was performed a Log-rank (Mantel-Cox) test with a Bonferroni correction for comparisons. (**D**) FACS blots of spleen, bone marrow and lymph node tissue showing the T-ALL that developed at day 182 post LCMV infection in a *Pax5*$^{-/+}$ mouse. Error bars indicate SEM. Source data are available online for this figure.

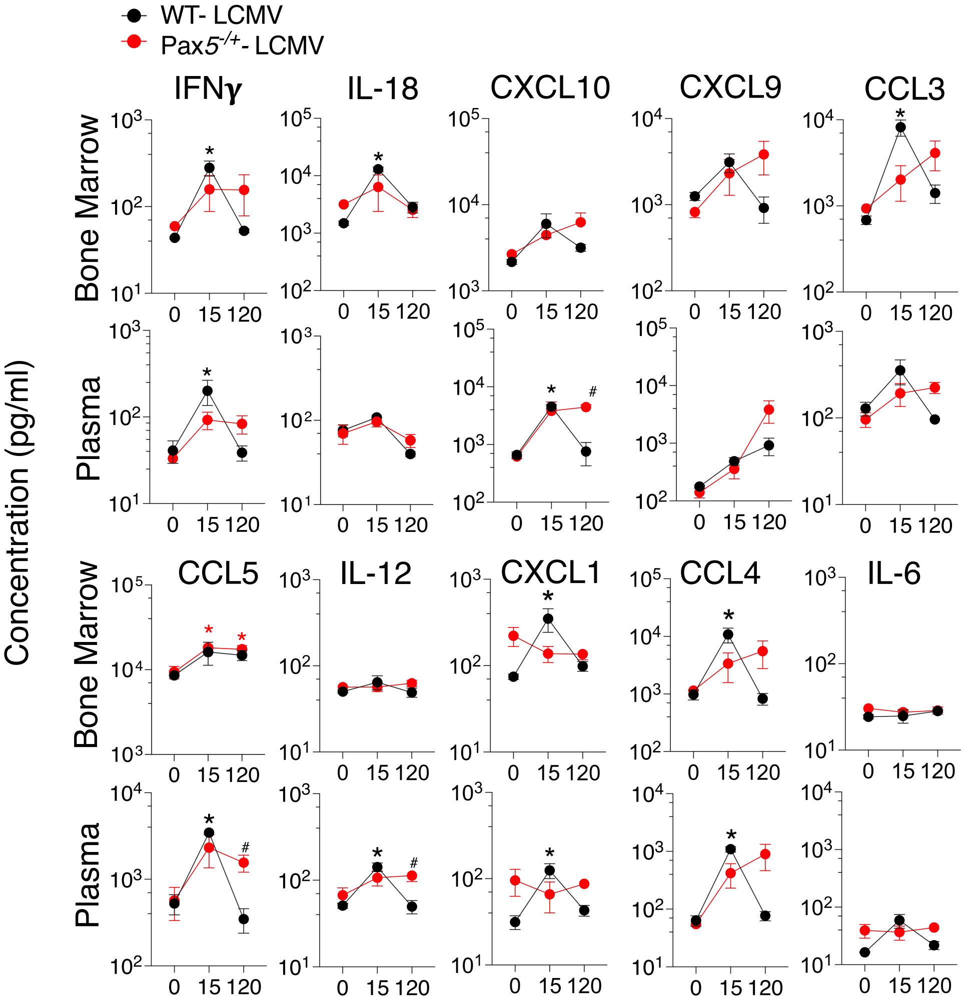

Figure EV2. Pax5 haploinsufficiency shapes distinct cytokine profiles in the plasma and bone marrow microenvironment in response to chronic infection.

Cytokine levels in the bone marrow and plasma were evaluated using the Procarta 19-Plex assay in uninfected naive controls (day 0), day 15 and day 120 post infection with 10^6 PFU of LCMV Docile in *Pax5*$^{-/+}$ and WT mice ($n = 3$ mice per group). Concentrations in pg/ml of detectible cytokines are shown. *As indicated in the figure represents significant differences relative to time point 0 within each genotype as determined by a one-way ANOVA with Dunnett's post hoc test. IFNγ (BM $P = 0.0036$, plasma $P = 0.045$), IL-18 ($P < 0.0001$), CXCL10 ($P = 0.0002$), CCL3 ($P = 0.004$), CCL5 (BM day 15 $P = 0.0105$, day 120 $P = 0.0155$, plasma $P < 0.0001$), IL-12 ($P = 0.0018$), CXCL1 (BM $P = 0.0358$, plasma $P = 0.0081$), CCL4 (BM $P = 0.0150$, plasma $P < 0.0001$). #As indicated in the figure represents significant differences between the *Pax5*$^{-/+}$ and WT group at a given time point as determined by a Student´s *t* test (unpaired, two-tailed). CXCL10 ($P = 0.003$), CCL5 ($P = 0.030$), IL-12 ($P = 0.028$). Error bars indicate SEM. Source data are available online for this figure.

 © The Author(s)

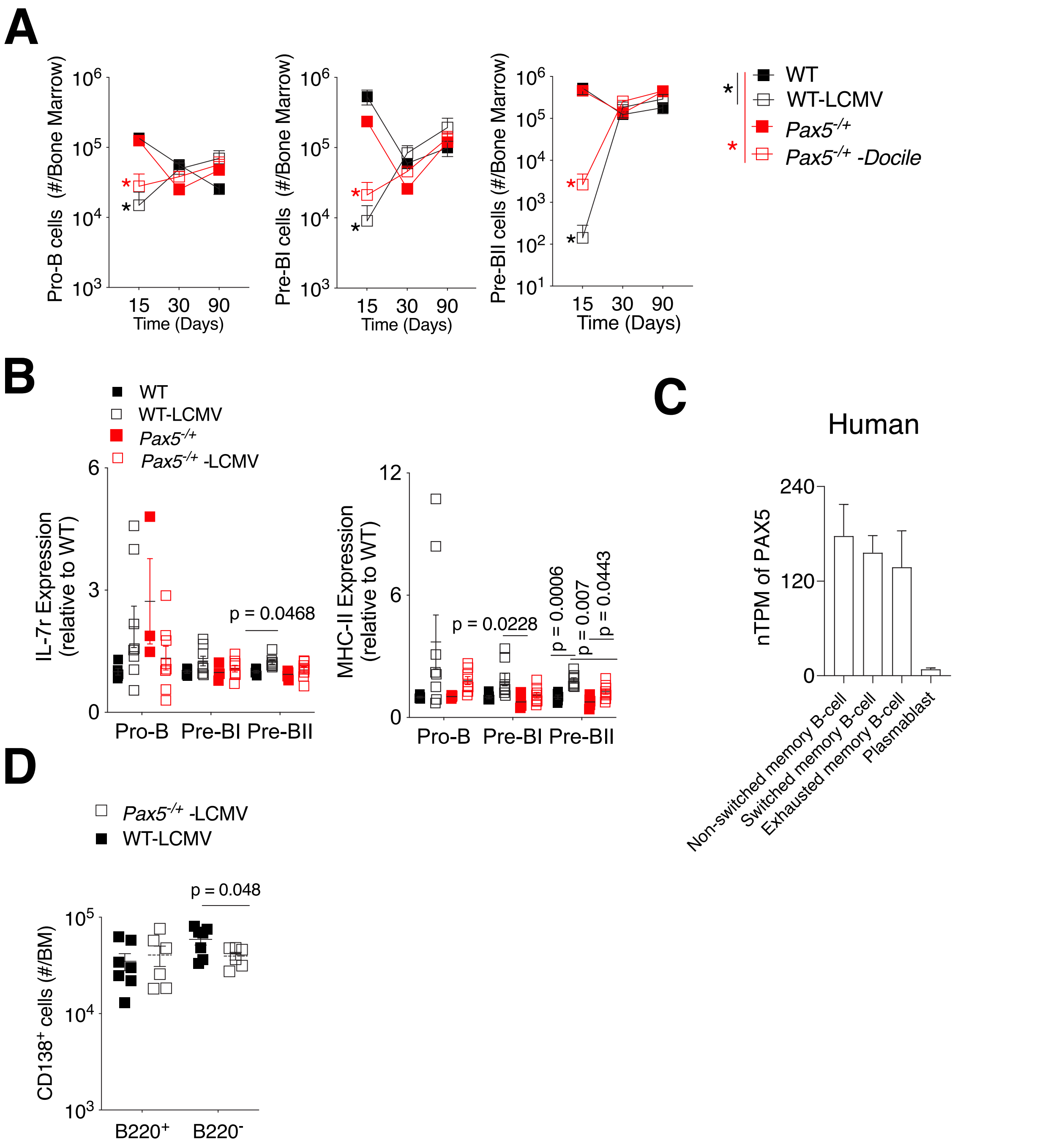

Figure EV3. Chronic LCMV infection affects early B cells populations in the bone marrow of *Pax5*$^{-/+}$ and WT hosts.

Pax5$^{-/+}$ and WT mice were intravenously infected with 10^6 PFU of LCMV Docile. (**A**) Pro-B, Pre-BI and Pre-BII cells were measured in the bone marrow at the indicated days post infection using FACS ($n \geq 3$ mice per group). (Pro-B, WT $P = 0.0007$ *Pax5*$^{-/+}$ $P = 0.0010$), (Pre-BI, WT $P = 0.001$ *Pax5*$^{-/+}$ $P = 0.0007$), (Pre-BII, WT $P = 0.0055$ *Pax5*$^{-/+}$ $P = 0.0037$). (**B**) Surface molecule MHC-II and IL-7r expression was measured on Pro-B, Pre-BI and Pre-BII cells in the bone marrow 90 days post infection ($n \geq 3$ mice per group). Statistical analyses for (**A**, **B**) were performed using a one-way ANOVA with a Tukey post hoc test. (**C**) PAX5 expression data in different human B-cell subsets was mined from The Human Protein Atlas, Monaco dataset ($n = 4$). (**D**) B220$^+$CD138$^+$ and B220$^-$CD138$^+$ numbers were evaluated in the bone marrow of WT ($n = 7$ mice per group) and *Pax5*$^{-/+}$ mice ($n = 6$) 120 days post infection using FACS analysis. Error bars indicate SEM. Statistical analysis was carried out using a Student´s *t* test (unpaired, two-tailed). Source data are available online for this figure.

© The Author(s)

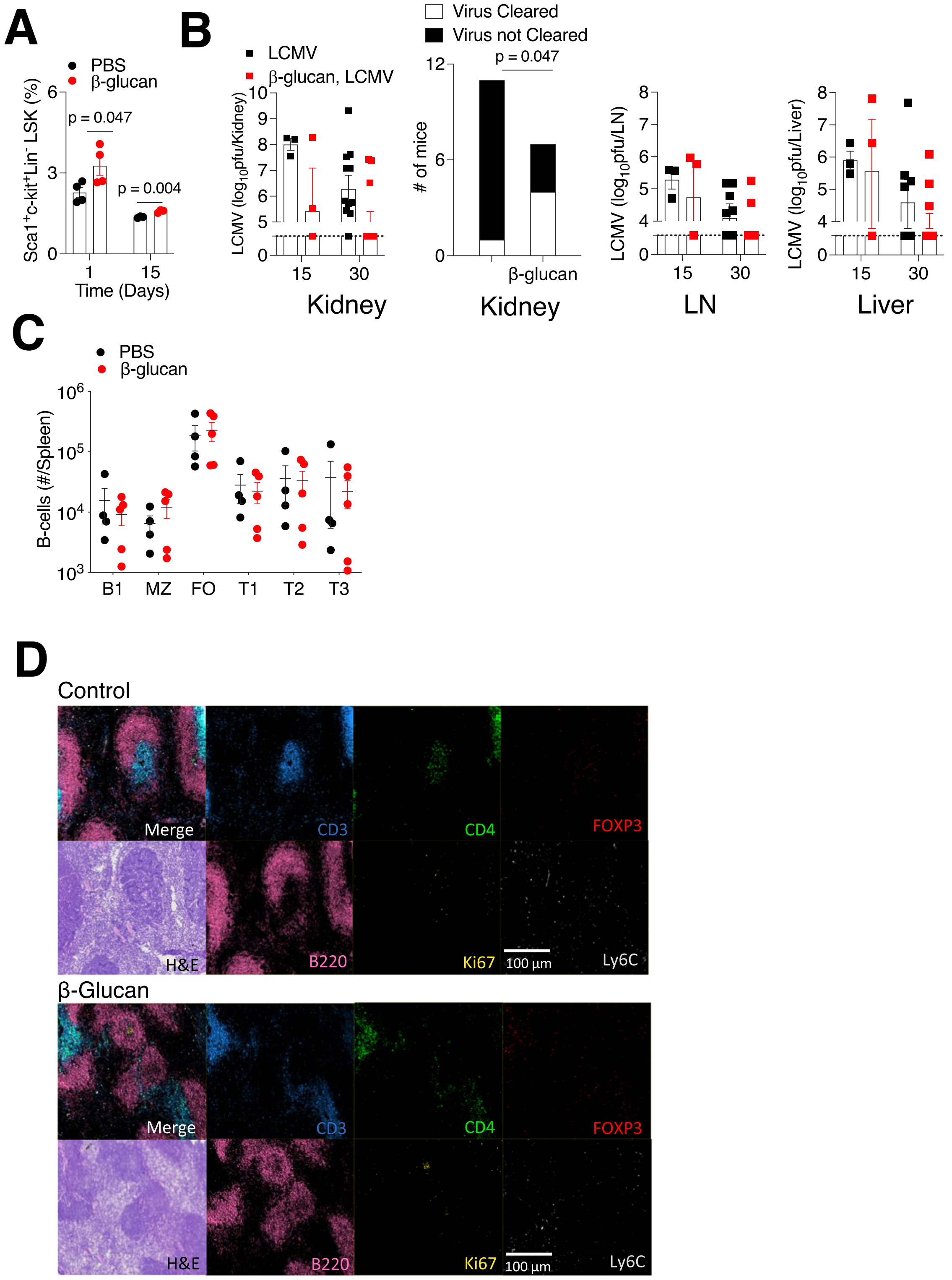
A
PBS
β-glucan
Sca1+c-kit+Lin- LSK (%)
p = 0.047
p = 0.004
Time (Days)
B
LCMV
β-glucan, LCMV
LCMV (log10pfu/Kidney)
Kidney
Virus Cleared
Virus not Cleared
# of mice
p = 0.047
β-glucan
Kidney
LCMV (log10pfu/LN)
LN
LCMV (log10pfu/Liver)
Liver
C
PBS
β-glucan
B-cells (#/Spleen)
B1
MZ
FO
T1
T2
T3
D
Control
Merge
CD3
CD4
FOXP3
H&E
B220
Ki67
100 μm
Ly6C
β-Glucan
Merge
CD3
CD4
FOXP3
H&E
B220
Ki67
100 μm
Ly6C

 © The Author(s)

Figure EV4. Immune training with β-glucan improves T-cell immunity following chronic LCMV infection.

(**A**) WT mice were injected with 1 mg of β-glucan and LSK ($Lin^{-}Sca\text{-}1^{+}c\text{-}KIT^{+}$) cells were measured in the bone marrow 24 h and 15 days post treatment ($n \geq 3$ mice per group). (**B**) WT mice were injected with 1 mg of β-glucan and 7 days later this was followed by infection with 10^6 PFU of LCMV Docile. LCMV virus titers were determined in the kidney, lymph node (LN) and liver at day 15 ($n = 3$ mice per group) and 30 ($n \geq 7$ mice per group) days post LCMV infection using plaque assay. Statistical analysis of the contingency table was done using a Fisher's exact test. (**C**) Numbers of B1, marginal zone (MZ), follicular (FO) and transitional (T1, T2 and T3) B cells were assessed in naive ($n = 4$ mice per group) and β-glucan ($n = 5$ mice per group) treated mice at day 30 post β-glucan treatment. (**D**) Splenic sections were stained with the indicated antibodies and detected using CO-Detection by indEXing (CODEX), scale bar indicates 100 µm; (a representative image of *n* of 5 (mice per group) is shown). Error bars indicate SEM. Unless otherwise stated, statistical analyses were performed using a Student´s *t* test (unpaired, two-tailed). Source data are available online for this figure.

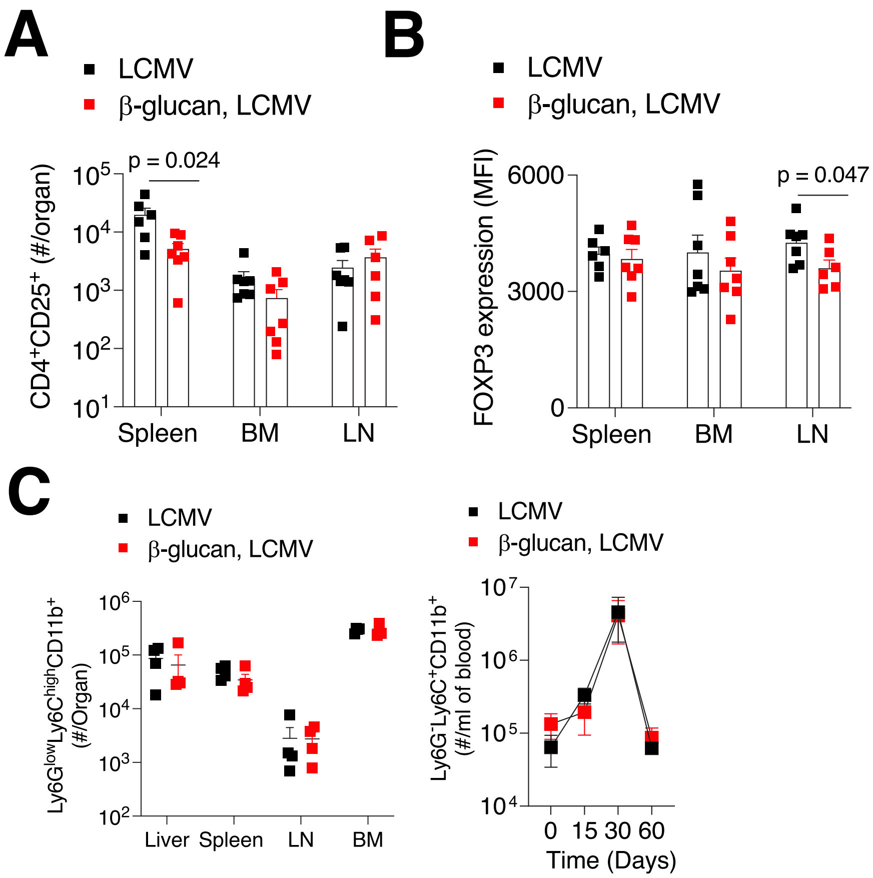

Figure EV5. Immune training with β-glucan affects immunosuppressive infiltrates following chronic LCMV infection.

WT mice were injected with 1 mg of β-glucan and 7 days later this was followed by infection with 10^6 PFU of LCMV Docile. (**A**) 30 days post infection, numbers of Treg's ($CD4^+CD25^+$) as well as (**B**) Treg FOXP3 expression were measured using FACS analysis in the spleen, bone marrow (BM) and lymph node (LN) of infected mice ($n \geq 6$ mice per group). (**C**) 30 Days post infection, frequencies of monocytes ($CD11B^+Ly6G^{low}Ly6C^{high}$) were measured using FACS analysis in the spleen, bone marrow (BM) and lymph node (LN), liver ($n = 4$ mice per group) and in the blood at the indicated time points ($n \geq 3$ mice per group). Error bars indicate SEM. Error bars indicate SEM. Statistical analyses were performed using a Student´s *t* test (unpaired, two-tailed). Source data are available online for this figure.

 © The Author(s)
